# Differential Co-Expression Network Analysis Reveals Key Hub-High Traffic Genes as Potential Therapeutic Targets for COVID-19 Pandemic

**DOI:** 10.3389/fimmu.2021.789317

**Published:** 2021-12-15

**Authors:** Aliakbar Hasankhani, Abolfazl Bahrami, Negin Sheybani, Behzad Aria, Behzad Hemati, Farhang Fatehi, Hamid Ghaem Maghami Farahani, Ghazaleh Javanmard, Mahsa Rezaee, John P. Kastelic, Herman W. Barkema

**Affiliations:** ^1^ Department of Animal Science, College of Agriculture and Natural Resources, University of Tehran, Karaj, Iran; ^2^ Nuclear Agriculture Research School, Nuclear Science and Technology Research Institute, Karaj, Iran; ^3^ Department of Animal and Poultry Science, College of Aburaihan, University of Tehran, Tehran, Iran; ^4^ Department of Physical Education and Sports Science, School of Psychology and Educational Sciences, Yazd University, Yazd, Iran; ^5^ Biotechnology Research Center, Karaj Branch, Islamic Azad University, Karaj, Iran; ^6^ Department of Medical Mycology, School of Medical Science, Tarbiat Modares University, Tehran, Iran; ^7^ Department of Production Animal Health, Faculty of Veterinary Medicine, University of Calgary, Calgary, AB, Canada

**Keywords:** systems biology, systems immunology, WGCNA, hub-high traffic genes, immunopathogenesis, therapeutic targets in infectious diseases, COVID-19 pandemic

## Abstract

**Background:**

The recent emergence of COVID-19, rapid worldwide spread, and incomplete knowledge of molecular mechanisms underlying SARS-CoV-2 infection have limited development of therapeutic strategies. Our objective was to systematically investigate molecular regulatory mechanisms of COVID-19, using a combination of high throughput RNA-sequencing-based transcriptomics and systems biology approaches.

**Methods:**

RNA-Seq data from peripheral blood mononuclear cells (PBMCs) of healthy persons, mild and severe 17 COVID-19 patients were analyzed to generate a gene expression matrix. Weighted gene co-expression network analysis (WGCNA) was used to identify co-expression modules in healthy samples as a reference set. For differential co-expression network analysis, module preservation and module-trait relationships approaches were used to identify key modules. Then, protein-protein interaction (PPI) networks, based on co-expressed hub genes, were constructed to identify hub genes/TFs with the highest information transfer (hub-high traffic genes) within candidate modules.

**Results:**

Based on differential co-expression network analysis, connectivity patterns and network density, 72% (15 of 21) of modules identified in healthy samples were altered by SARS-CoV-2 infection. Therefore, SARS-CoV-2 caused systemic perturbations in host biological gene networks. In functional enrichment analysis, among 15 non-preserved modules and two significant highly-correlated modules (identified by MTRs), 9 modules were directly related to the host immune response and COVID-19 immunopathogenesis. Intriguingly, systemic investigation of SARS-CoV-2 infection identified signaling pathways and key genes/proteins associated with COVID-19’s main hallmarks, e.g., cytokine storm, respiratory distress syndrome (ARDS), acute lung injury (ALI), lymphopenia, coagulation disorders, thrombosis, and pregnancy complications, as well as comorbidities associated with COVID-19, e.g., asthma, diabetic complications, cardiovascular diseases (CVDs), liver disorders and acute kidney injury (AKI). Topological analysis with betweenness centrality (BC) identified 290 hub-high traffic genes, central in both co-expression and PPI networks. We also identified several transcriptional regulatory factors, including *NFKB1*, *HIF1A*, *AHR*, and *TP53*, with important immunoregulatory roles in SARS-CoV-2 infection. Moreover, several hub-high traffic genes, including *IL6*, *IL1B*, *IL10*, *TNF*, *SOCS1*, *SOCS3*, *ICAM1*, *PTEN*, *RHOA*, *GDI2*, *SUMO1*, *CASP1*, *IRAK3*, *HSPA5*, *ADRB2*, *PRF1*, *GZMB*, *OASL*, *CCL5*, *HSP90AA1*, *HSPD1*, *IFNG*, *MAPK1*, *RAB5A*, and *TNFRSF1A* had the highest rates of information transfer in 9 candidate modules and central roles in COVID-19 immunopathogenesis.

**Conclusion:**

This study provides comprehensive information on molecular mechanisms of SARS-CoV-2-host interactions and identifies several hub-high traffic genes as promising therapeutic targets for the COVID-19 pandemic.

## Introduction

Coronavirus disease 2019 (COVID-19) is an infectious disease that was first reported in Wuhan, China in December 2019 (1). This viral pneumonia spread rapidly, with the World Health Organization (WHO) declaring a global pandemic on March 11, 2020 (2). COVID-19, caused by a single-stranded RNA beta coronavirus called severe acute respiratory syndrome coronavirus 2 (SARS-CoV-2), affects the lower respiratory tract in humans (3). As of 1 October 2021, SARS-CoV-2 has infected 233,503,524 people and caused 4,777,503 deaths worldwide (https://covid19.who.int/table). Primary symptoms of SARS-CoV-2 infection include fever, cough, shortness of breath, and pneumonia (4, 5). The recent emergence of COVID-19 and inadequate knowledge of infection progress, molecular mechanisms involved in the disease, interactions between SARS-CoV-2 and the host, and their relationship to disease outcomes limit our ability to develop effective treatments for infected patients. Therefore, understanding molecular/immunological mechanisms underlying the various clinical symptoms of COVID-19 are deemed critical in development of potential therapeutic strategies (6, 7).

Detecting changes in gene expression in relevant tissues during SARS-CoV-2 infection through various functional genomic methods (e.g., microarrays and RNA-sequencing-based transcriptomics) can increase understanding of molecular mechanisms involved in COVID-19, plus host-pathogen interactions (8–10). Transcriptomic studies in COVID-19 patients used lung epithelial cells, nasopharyngeal swabs, bronchoalveolar lavage fluid (9, 11–14), or peripheral blood mononuclear cells (PBMCs) (6, 8, 15). However, differential gene expression analysis focuses more on individual effects of genes (16), whereas genes interact in complex biological gene networks (17). Therefore, investigation of gene or protein interactions at the systems level should elucidate dynamics of SARS-CoV-2 infection and molecular mechanisms responsible for COVID-19.

Recent computational methods of systems biology, including network analysis and machine learning, are suitable for analyzing omics techniques such as high throughput RNA-sequencing (RNA-seq) at the systemic level (18). Weighted gene co-expression network analysis (WGCNA) is a systems biology method to identify clusters (modules) of highly correlated genes, candidate biomarkers, and therapeutic targets (19); it has been used in various human infectious diseases, e.g., Influenza (20), Tuberculosis (21, 22), Hepatitis B,C (23–25), HIV (26), and various cancers (27–30). Additionally, in some studies using the module-trait relationships approach of WGCNA, modules related to clinical traits of COVID-19 were identified at various stages (31–33). WGCNA has a unique network-based approach called module preservation analysis (34), based on topological changes across conditions, e.g., comparison of samples from healthy versus diseased persons. Simply stated, differences in connectivity patterns and network density between healthy and disease samples as reference and test sets, respectively, indicate a disease-induced perturbation of the network (34). Therefore, non-preserved modules between healthy and disease samples are important candidates for biological investigation of a disease such as COVID-19. The module preservation approach of WGCNA is valuable for differential network analysis (17, 34) and has been successfully used for several human (35–37) and animal (16, 38) diseases.

Combining high-throughput technologies with computational systems biology methods such as WGCNA provides new opportunities to better understand molecular mechanisms responsible for diseases such as COVID-19 (39). To the best of our knowledge, this is the first differential co-expression network analysis on COVID-19, with the following purposes (1): Combining RNA-seq data and WGCNA to identify co-expression modules in healthy samples as the reference set (2); Module preservation analysis to detect non-preserved modules between healthy and COVID-19 (as test set) samples (3); Module-trait relationships analysis to identify significant highly-correlated modules with disease severity (4); Functional enrichment analysis of non-preserved modules for biological assessment and understanding molecular regulatory mechanisms behind COVID-19 (5); Identification of hub genes and potential transcription factors (TFs) in non-preserved modules; and (6) Extraction of protein-protein interaction (PPI) networks, based on hub genes of candidate modules for topological analysis, using betweenness centrality (BC) to identify hub genes with the highest BC score (hub-high traffic genes) as vital bridges for information transfer inside modules.

## Materials and Methods

### Gene Expression Datasets

RNA-sequencing (RNA-seq) raw reads of COVID-19 patients and healthy persons were obtained from the Gene Expression Omnibus (GEO) database at the National Center for Biotechnology Information (NCBI; accession number GSE152418) and the European Genome-phenome Archive (EGA; accession number EGAS00001004571). These datasets included 87 samples of PBMCs from healthy (n=17), mild (n=33) and severe (n=37) groups. An Illumina NovaSeq 6000 platform was used to generate 101-bp single-end reads, as detailed in the original reports (6, 7).

### RNA-Seq Data Analysis

FastQCsoftware version 0.11.9 (https://www.bioinformatics.babraham.ac.uk/projects/fastqc/) was used for quality control of raw reads. Next, adapter sequences, low-quality reads, and bases were trimmed by Trimmomatic software (Version 0.39) (40) using the following criteria: ILLUMINACLIP: Adapter.fa:2:30:10, LEADING:20, SLIDINGWINDOW:6:20, TRAILING:20, and MINLEN:50. After obtaining clean reads, their quality was verified with FastQC software to confirm improvements. Then, clean reads were aligned to the latest human reference genome (GRCh38) using Hisat2 software version 2.2.1 with default parameters (41). Python script HTSeq-count (42) version 0.13.5 was used to count uniquely mapped reads to annotated genes, based on the ENSEMBL human GTF file (release 104). Finally, all count files were merged into a table and a raw expression matrix created and normalized to log-counts per million (log-CPM) using the voom function of the limma R package (Version 3.46.0) (43, 44). To prevent sampling noise caused by low-expressed or low-variance genes, only genes with expression ≥ 1 count per million reads (CPM) in at least 5 samples and standard deviation > 0.25 were selected for downstream analyses.

### Weighted Gene Co-Expression Network Analysis (WGCNA)

Based on the assumption that non-preserved modules between healthy controls and COVID-19 patients may explain biological behavior of COVID-19 and increase understanding of molecular mechanisms responsible for SARS-CoV-2 infection, healthy samples were selected as a reference set for construction of a weighted gene co-expression network and modules detection. In this study, a signed weighted gene co-expression network was constructed according to the standard procedure of WGCNA R package Version 1.70 (19). In general, signed networks provide a better understanding of molecular regulatory mechanisms at the systemic level, facilitating better separation of modules in terms of biological performance (19, 45). Initially, as WGCNA is very sensitive to outliers, the adjacency function of the WGCNA R package was used to construct distance-based adjacency metrics of samples; those with a standardized connectivity score < −2.5 were considered an outlier and removed. To ensure a scale-free network, pickSoftThreshold function of the WGCNA R package was used to identify the soft thresholding power *β* value. Moreover, since a Pearson correlation is susceptible to outliers, bi-weight mid-correlation were used to calculate pairwise correlations between genes, as it is more robust to outliers (46, 47). Therefore, a weighted adjacency matrix was constructed using the bi-weight mid-correlation coefficient at *β* = 12 as a soft thresholding power and subsequently transformed into a topological overlap matrix (TOM). Next, an average linkage hierarchical clustering analysis was performed based on the TOM dissimilarity (1-TOM) and modules were detected through a dynamic hybrid tree cutting algorithm. Finally, modules with very similar expression profiles or highly correlated eigengenes were merged. All of the above steps were performed by the 1-step network construction and module detection blockwiseModules function of the WGCNA R package with the following options: power = 12, networkType = “signed”, TOMType = “signed”, maxBlockSize = 15000, corType = “bicor”, reassignThreshold = 0, mergeCutHeight = 0.25, and minModuleSize = 30.

### Module Preservation Analysis

To investigate whether the topological structure of modules changed between healthy (reference set) and COVID-19 (test set) samples, the modulePreservation function of the WGCNA R package was used for preservation analysis. In this step, 2 composite preservation statistics, Zsummary and medianRank, were investigated based on 200 random permutations. Modules with a higher value of Zsummary represent high preservation across conditions. However, Zsummary is highly dependent on module size, and as it increases, Zsummary also increases, whereas medianRank is independent of module size and unlike Zsummary, modules with low medianRank values indicate strong preservation across various conditions (34). Modules with values Zsummary > 10 and medianRank < 8 indicates high preservation between groups (34). Therefore, in this study, modules with values of Zsummary ≤ 10 or medianRank ≥ 8 were considered non-preserved modules.

### Module-Trait Relationships Analysis

To identify significant highly-correlated modules with disease severity of COVID-19, 25 mild and 29 severe COVID-19 patients were used for module-trait relationships (MTRs) analysis. Because the gene co-expression analysis is very sensitive to outliers, the distance-based adjacency metrics of samples was calculated and samples with a standardized connectivity < −2.5 were removed, considered as an outlier. In addition, samples and genes with > 50% missing entries and genes with zero variance were identified and excluded from WGCNA analysis (35). Briefly, a correlation matrix of expression values was constructed using pairwise bi-weight mid-correlation coefficients between all pairs of genes across the selected samples. In other words, the genes in a module share strong interconnectedness (36). Finally, average linkage hierarchical clustering analysis was performed by the topological overlap-based dissimilarity matrix (1-TOM) as input, and modules were identified by dynamic hybrid tree cutting algorithm. Then, the modules with the highly correlated eigengenes were merged. The above steps were performed using automatic, one-step network construction and module detection function “blockwiseModules” of the WGCNA R package. Next, in order to identify the COVID-19-related modules, the correlation between the disease severity of COVID-19 and module eigengenes was taken using Pearson correlation coefficient.

### Functional Enrichment Analysis of the Non-Preserved Modules

Enrichr online web tool (48) was used to analyze Gene Ontology (GO) and Kyoto Encyclopedia of Genes and Genomes (KEGG) pathways for non-preserved modules. Thus, all genes in each non-preserved module were investigated for functional characterization; only terms with a threshold of adj *p* value < 0.05 were deemed significant (*p* values corrected by the Benjamini-Hochberg method).

### Hub Genes and TFs Detection in Non-Preserved and Correlated Modules

In complex biological networks, intramodular genes with the highest degree of connections (hub genes) have more biological relevance in association with a disease (49–52). Module memberships (MM) or eigengene-based connectivity *k_ME,_* a criterion indicating the relationship of a gene with the relevant module, is calculated as the correlation between gene expression profile and module eigengenes (the first principal component of the expression profile for a module) by the WGCNA R package. Genes with higher MM values have a more significant relationship with biological performance of the respective module and act as central genes in that module (19, 53, 54). Therefore, genes with *k_ME_* ≥ 0.7 were selected as intramodular hub genes in modules. Next, for construction of protein-protein interaction (PPI) networks, identified hub genes in each module were subjected to the search tool for retrieval of interacting genes (STRING) database (55). Additionally, transcriptional regulatory factors within modules were identified using a set of human transcription factors extracted from the HumanTFDB database (56).

### Identification of Hub-High Traffic Genes and Network Visualization

PPI networks derived from hub genes of non-preserved modules from the previous step were used for topological analyses with the betweenness centrality (BC) measure. In connected networks, BC is a general measure of centrality, based on the number of shortest paths between every 2 other genes that pass through a certain gene, indicating influence over information transfer between genes in modules (57–61). Genes with the highest BC scores, termed high traffic genes, have a central role in association with the biological behavior of the respective module (36, 62). In this study, we used 2 methods to calculate BC scores in the hub gene-base PPI networks from modules. The first method was to calculate BC scores through cytoHubba (a cytoscape plugin) Version 0.1, using the Betweenness algorithm (63), whereas the second method was to calculate BC scores through Igraph (Version 1.2.6) R package (https://cran.r-project.org/web/packages/igraph/). Finally, overlapped genes with the highest BC score between these 2 methods were selected and the top 50 hub genes in modules with a size of ≥ 450 and the top 10 hub genes in modules with a size of ≤ 180 in terms of BC score were considered hub high-traffic genes. Additionally, COVID-19 biologically related modules were visualized using Cytoscape Version 3.8.2 (64).

## Results

### RNA-Seq Data Analysis

**Figure 1:**
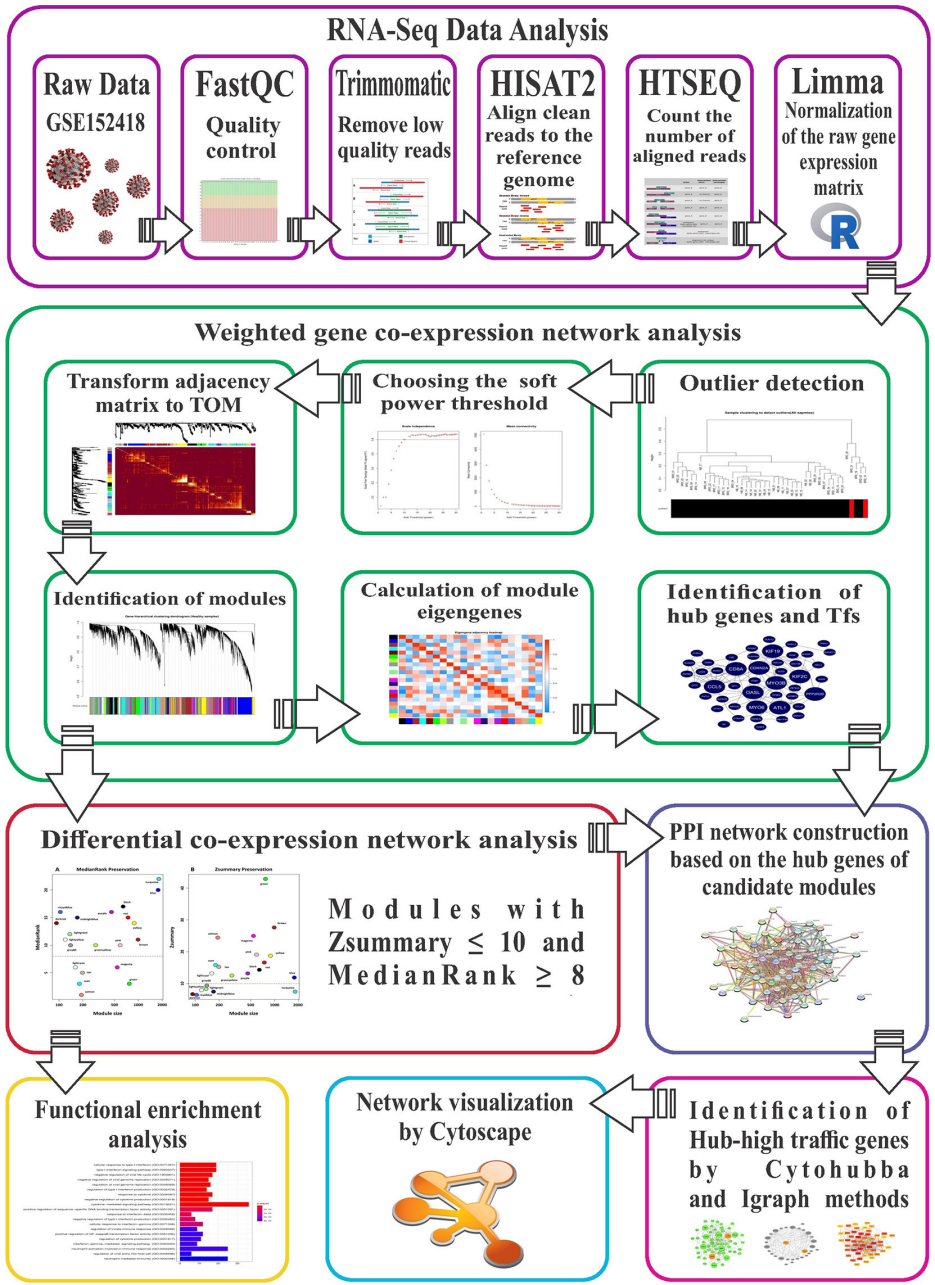
The stringent step-by-step pipeline of the RNA-seq data analysis and differential co-expression network approach in this study.

The stringent step-by-step workflow of the differential co-expression network analysis used in this study is shown (**Figure 1** **)**. Briefly, RNA-seq data contained 87 samples (17 healthy and 70 COVID-19 patients) of PBMCs, with an average of 23 million reads per sample. Details regarding RNA-seq data analysis and preprocessing are in **Supplementary Table 1**. After normalization and filtering of low expressed and low variance genes, 11663 genes remained for weighted gene co-expression network construction. The normalized and filtered gene expression matrix of healthy controls and COVID-19 patients is in **Supplementary Table 2**.

### Weighted Gene Co-Expression Network Construction

**Figure 2:**
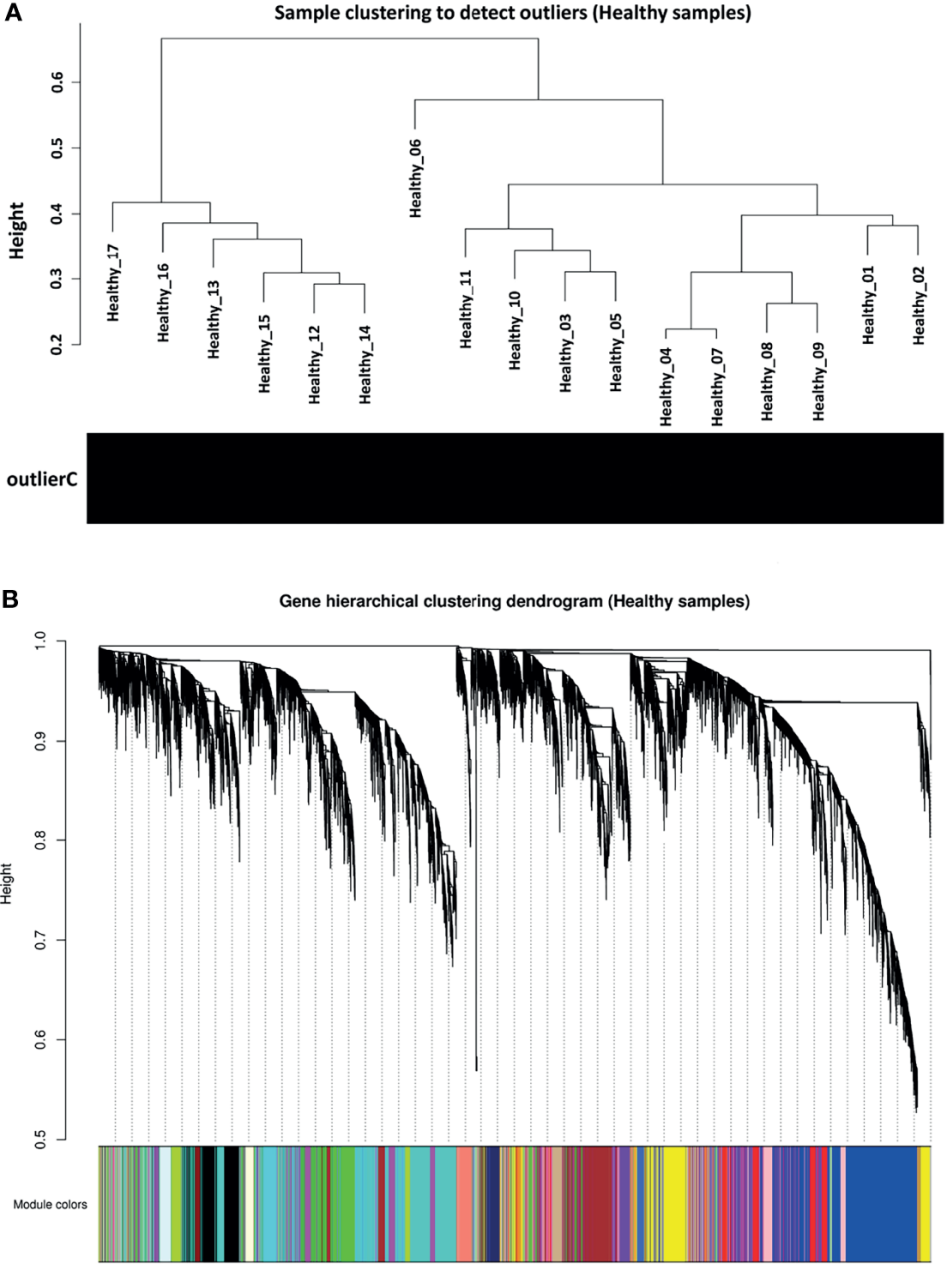
Preprocessing of weighted gene co-expression network analysis. **(A)** Sample clustering to detect outliers in healthy samples as reference set. All samples had a standardized connectivity score > −2.5. **(B)** Gene hierarchical clustering dendrogram of 21 detected modules based on a dissimilarity (1-TOM) measure. Branches represent modules that are marked with a specific color. The y-axis represents the co-expression distance, the x-axis represents genes and the grey module represents background genes.

Before co-expression network construction and module detection, distance-based adjacency metrics of samples were constructed to identify outliers and prevent their negative influence on network analysis. All samples had a standardized connectivity score > −2.5, with no outliers (**Figure 2A**). Weighted gene co-expression network was constructed at *β* = 12, representing a scale free topology fitting index (*R^2^*) ≥ 0.80. In total, 21 co-expression modules (average of 525 genes) and 647 background genes (grey module) were identified in healthy samples (reference set) through hierarchical clustering analysis and dynamic hybrid tree cutting algorithm. Moreover, each module as a branch of the hierarchical clustering dendrogram was labeled with a specific color by the WGCNA R package (**Figure 2B**). The turquoise module with a size of 1818 and the darkred module with 93 genes were the largest and smallest modules, respectively. Complete module information is provided in **Supplementary Table 3**.

### Module Preservation Analysis

**Figure 3:**
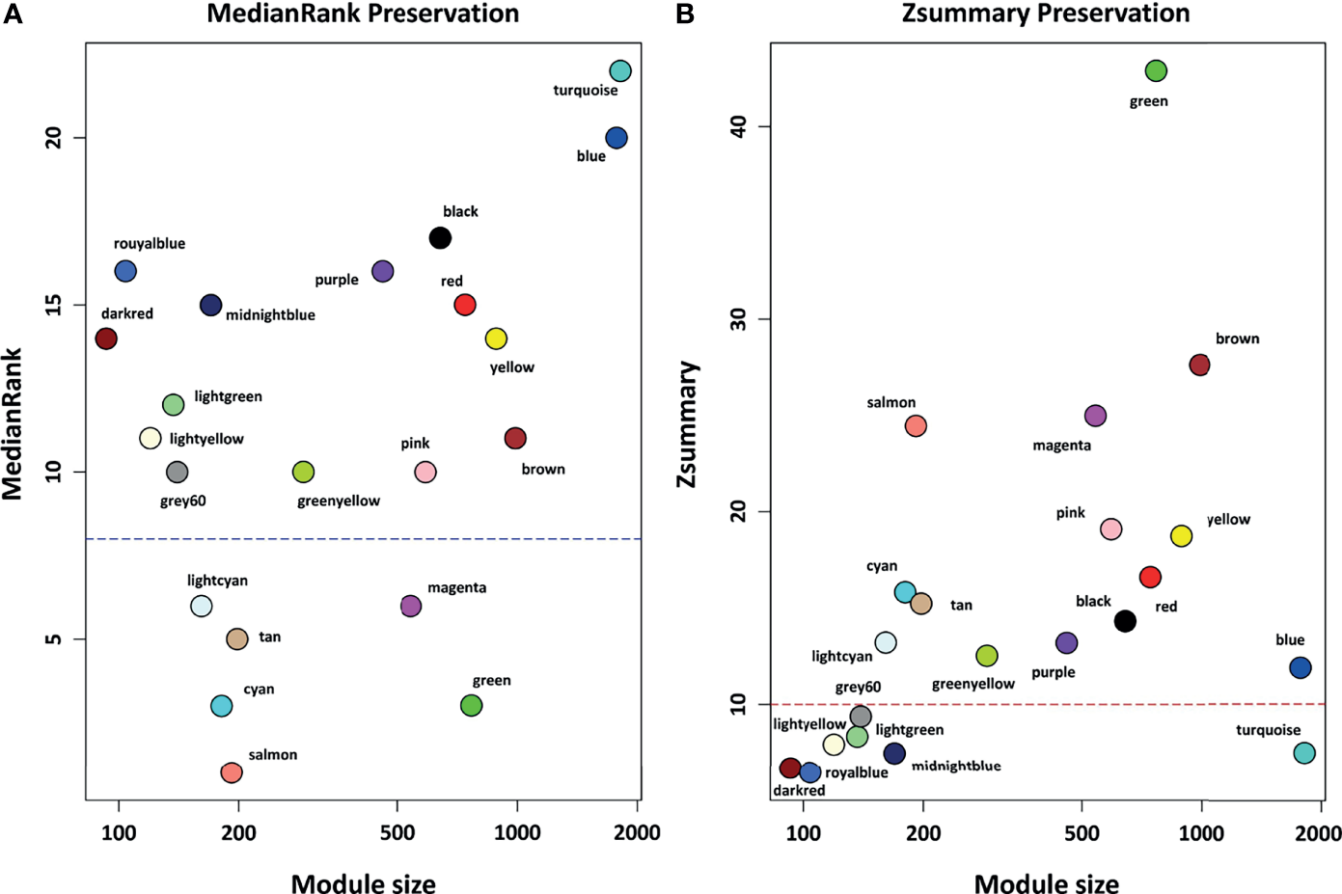
The preservation status of the respective modules. **(A)** MedianRank preservation results. The y axis represents medianRank values and x axis represent module size. Each point with a specific color represents the corresponding module. **(B)** Zsummary preservation results. The y axis represents Zsummary values and x axis represents the module size. Each point with a specific color represents the corresponding module. Modules with medianRank ≥ 8 (the blue dashed line) or Zsummary ≤ 10 (the red dashed line) were considered non-preserved between healthy controls and COVID-19 samples.

Based on module preservation analysis, among identified modules, the topological structure of 6 modules including salmon (medianRank = 1; Zsummary = 24), cyan (medianRank = 3; Zsummary = 16), green (medianRank = 3; Zsummary = 43), tan (medianRank = 5; Zsummary = 15), lightcyan (medianRank = 6; Zsummary = 13), and magenta (medianRank = 6; Zsummary = 25) was highly-preserved between healthy and COVID-19 samples (**Figure 3**). Moreover, according to our assumption, connectivity patterns and network densities of the other 15 modules were changed under COVID-19 conditions (**Figure 3**). Among the 15 non-preserved modules, the turquoise module (medianRank = 22; Zsummary = 7.4) had the highest degree of change in the topological structure affected by SARS-CoV-2 infection. More information regarding preservation status of all modules is available in **Supplementary Table 4**.

### Functional Enrichment Analysis of the Non-Preserved Modules

**Figure 4:**
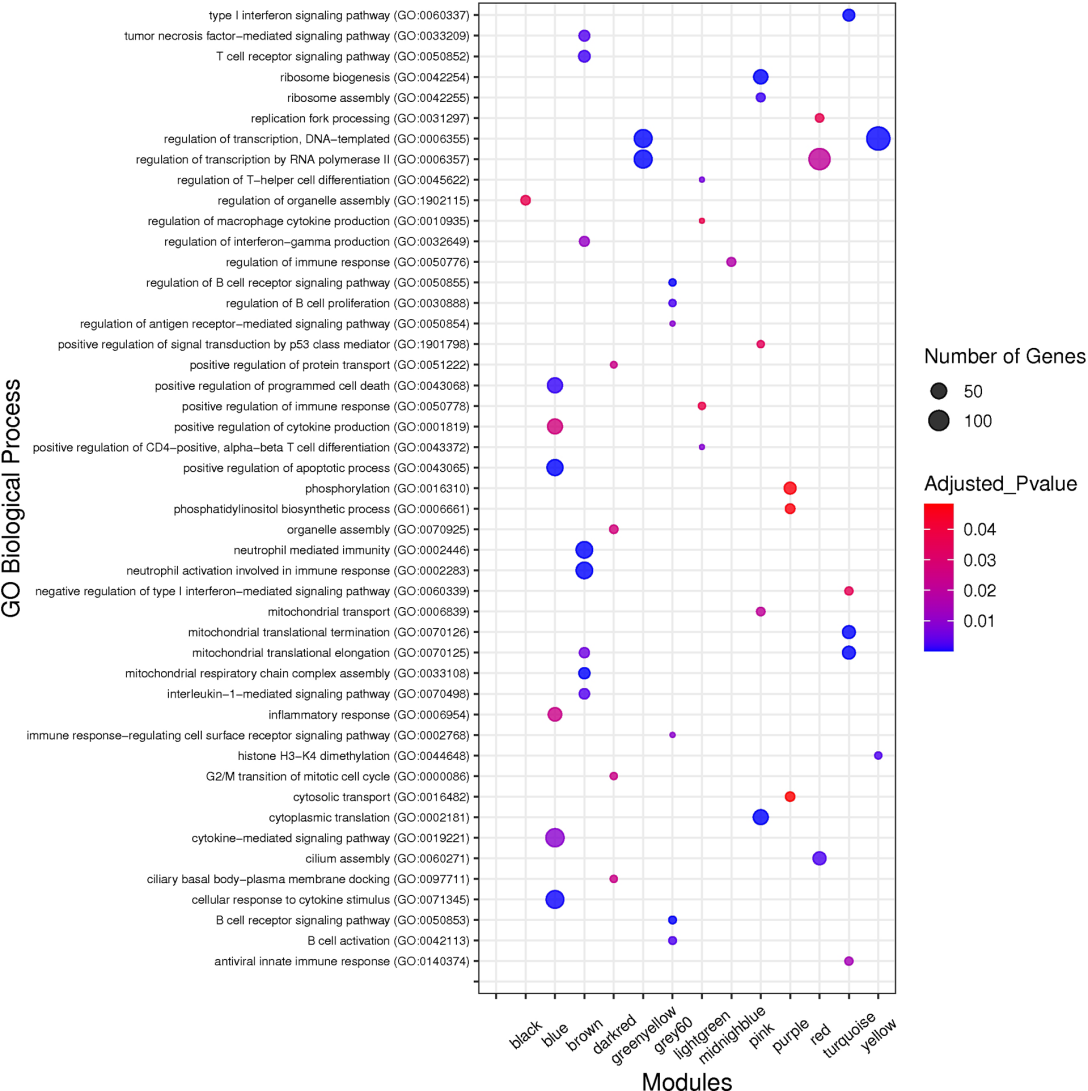
The top GO biological processes of the non-preserved modules. The y axis and the x axis represent significant enriched GO terms and module name, respectively. Color and size of each point represent adjusted *p* value and number of genes for each term, respectively.

To identify biological processes associated with non-preserved modules, GO analysis was performed and a total of 320 biological processes were significantly enriched in the 13 non-preserved modules including blue, brown, grey60, lightgreen, midnightblue, pink, purple, turquoise, black, darkred, greenyellow, red, and yellow. In the other 2 non-preserved modules, including lightyellow and royalblue, no biological process was significantly enriched. Conversely, KEGG pathways analysis identified 97 significant terms in 10 non-preserved modules (blue, brown, grey60, lightgreen, lightyellow, pink, purple, greenyellow, red, and yellow) as well as no enriched term in the other 5 non-preserved modules (midnightblue, turquoise, black, darkred, and royalblue). Additionally, among the non-preserved modules, the blue module had the most significant enriched terms with 148 and 47 biological processes and KEGG pathways, respectively. Complete information regarding functional enrichment analysis is provided in **Supplementary Table 5**. Based on functional enrichment analysis, 9 non-preserved modules including blue, brown, grey60, lightgreen, lightyellow, midnightblue, pink, purple, and turquoise were related to the host immune response and SARS-CoV-2 infection. Among these 9 candidate non-preserved modules, the blue module had the most biological associations with the immunopathogenesis of COVID-19. The top biological processes of non-preserved modules are shown in **Figure 4**.

### TFs, Hub TFs, Hub Genes, and Hub-High Traffic Genes Identification in Non-Preserved Modules

**Figure 5:**
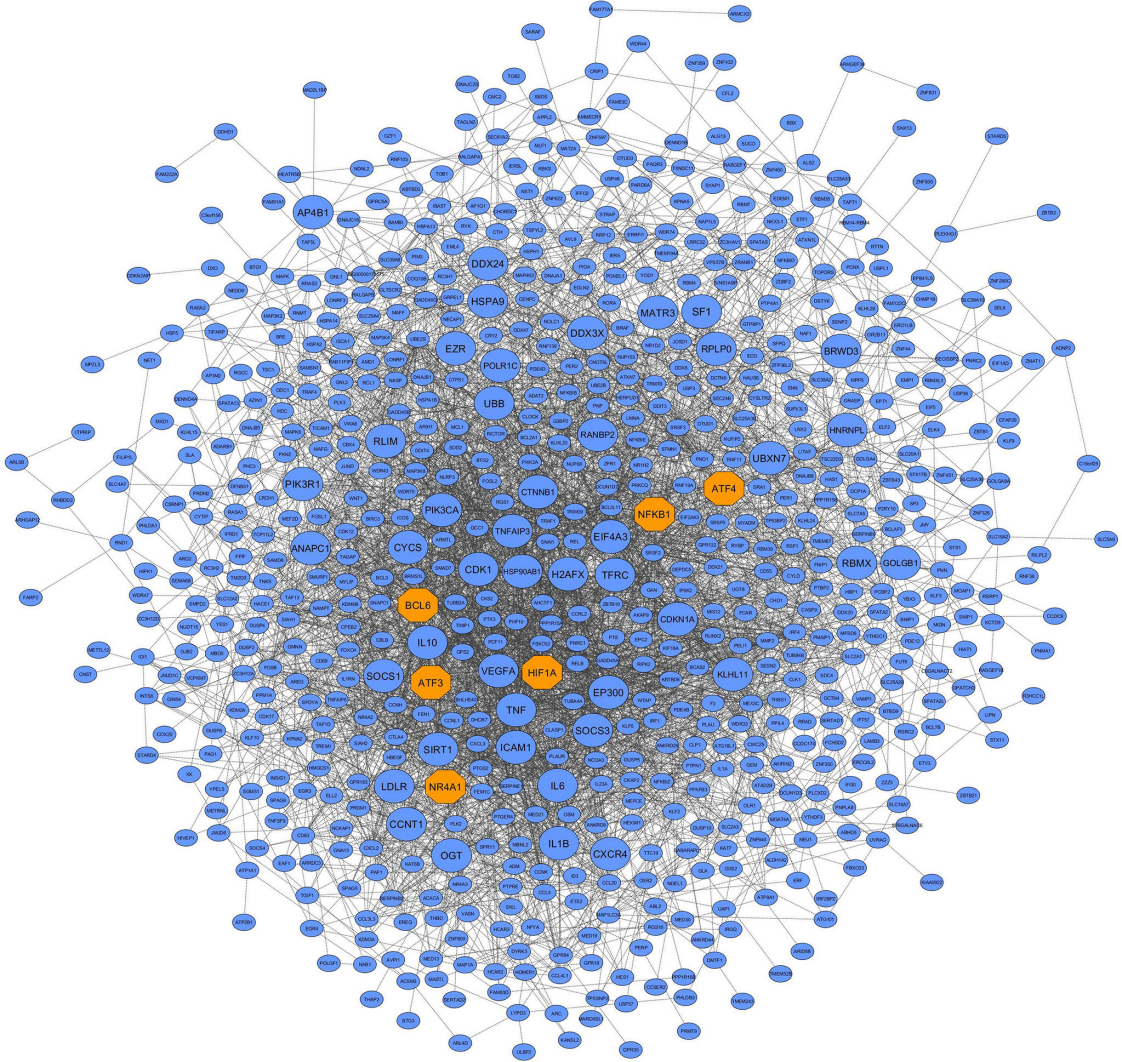
PPI network based on the co-expressed hub genes of the blue module. This module had the most biological associations with the immunopathogenesis of COVID-19. Large circles and orange octagons represent hub-high traffic genes and TFs, respectively.

Here, the differential co-expression network approach and functional enrichment analysis revealed 9 candidate non-preserved modules with a: 1) change in topological structure due to COVID-19; and 2) biological relation to SARS-CoV-2 infection. Using the MM criterion to assess these modules, a total of 881, 369, 85, 62, 59, 64, 251, 253, and 555 intramodular hub genes were identified in the blue, brown, grey60, lightgreen, lightyellow, midnightblue, pink, purple, and turquoise modules, respectively. The complete list of the hub genes of the non-preserved modules is provided in **Supplementary Table 6**. In addition, based on co-expressed hub genes, the PPI network of the blue module is shown in **Figure 5**. Additionally, based on human transcriptional regulatory factors extracted from the HumanTFDB database, a total of 183, 33, 12, 14, 5, 10, 34, 18, and 131 TFs were detected in the blue, brown, grey60, lightgreen, lightyellow, midnightblue, pink, purple, and turquoise modules, respectively. Complete information of TFs detected in non-preserved modules are in **Supplementary Table 7**. Furthermore, among detected hub genes, we identified 110, 5, 8, 8, 2, 5, 16, 11, and 49 TFs (hub TFs) in the blue, brown, grey60, lightgreen, lightyellow, midnightblue, pink, purple, and turquoise modules, respectively (**Supplementary Table 8**). Moreover, the identified hub-high traffic genes in the 9 candidate non-preserved modules along with their BC scores obtained by both cytoHubba and Igraph methods are presented in **Table 1** and **Supplementary Table 9**. These genes were highly connected intramodular hubs in the 9 candidate co-expression modules and they were also central genes for information transfer within the hub-gene based PPI networks of their respective modules. Therefore, hub-high traffic genes are considered important candidates for prognostic and therapeutic targets for COVID-19. Additionally, PPI networks of the 9 candidate non-preserved modules are in **Supplementary Figure 1**.

**Table 1:** List of the identified hub-high traffic genes in the 9 candidate non-preserved modules.

Module								
Blue	Grey60	Brown	Lightgreen	Pink	Lightyellow	Purple	Midnightblue	Turquoise
*EP300*	*CD19*	*RBX1*	*GLUL*	*HSP90A*	*ERBB2*	*MAPK1*	*CDKN2A*	*TP53*
*UBB*	*IGLL5*	*PSMB2*	*TRIB1*	*A1*	*ADRB2*	*CLTC*	*OASL*	*UTP14A*
*CDK1*	*CD79B*	*PSMA3*	*HSPA5*	*RPS27A*	*NCAM1*	*CUL2*	*CCL5*	*PXN*
*VEGFA*	*CD79A*	*PSMA6*	*VIM*	*EZH2*	*GZMB*	*CMTM6*	*MYO3B*	*RUVBL2*
*TNF*	*BLK*	*PTEN*	*ARF4*	*CEP290*	*PRF1*	*VPS26A*	*MYO6*	*MRTO4*
*CTNNB1*	*PNOC*	*VPS29*	*PLEK*	*NPM1*	*TBX21*	*ATP6V1D*	*KIF19*	*PCBP1*
*IL6*	*SPIB*	*NDUFAB1*	*ACSL1*	*HNRNPA1*	*GZMA*	*PJA2*	*PPP2R2B*	*POLR2H*
*SIRT1*	*CD22*	*SNRPE*	*TUBA1B*	*PPP2CA*	*NKG7*	*TXNRD1*	*KIF2C*	*BYSL*
*NFKB1*	*POU2AF*	*PSMA4*	*IRAK3*	*POLR2K*	*KLRD1*	*RAB1A*	*CD8A*	*PRKCD*
*TFRC*	*1*	*TXN*	*AHR*	*TPT1*	*B3GAT1*	*TMED7*	*ATL1*	*KEAP1*
*RANBP2*	*TCF3*	*PTGES3*		*EEF1A1*		*DICER1*		*IMP3*
*HSP90A*		*RHOA*		*RPL5*		*RAB5A*		*CEBPA*
*B1*		*ATP5C1*		*EEF1B2*		*TRIP12*		*ICT1*
*UBXN7*		*CANX*		*HSPD1*		*HSP90B1*		*RPL26L1*
*IL10*		*VAMP7*		*DDX5*		*SNRPG*		*ARHGAP30*
*CYCS*		*B2M*		*HSPE1*		*DNAJC10*		*PTPN6*
*IL1B*		*GDI2*		*ISCU*		*MAGT1*		*WDR18*
*EZR*		*SNRPD1*		*RPS3A*		*LYPLA1*		*DDX28*
*RPLP0*		*UBE2L3*		*IFNG*		*HNRNPH2*		*RFC2*
*HIF1A*		*TXNL1*		*CD59*		*CYB5R4*		*HGH1*
*CDKN1A*		*CCT8*		*RRM1*		*ADAM10*		*PPARA*
*HSPA9*		*SUMO1*		*RPL21*		*CREB1*		*MRPS18B*
*EIF4A3*		*UBE2D1*		*RPGR*		*TXNDC16*		*COASY*
*CCNT1*		*MCTS1*		*CCDC14*		*VBP1*		*MCM3*
*ATF4*		*NDUFC2*		*PTPRC*		*SNX2*		*TNFRSF1A*
*PIK3CA*		*PSMC2*		*LDHA*		*STX12*		*PFDN6*
*PIK3R1*		*RAB11A*		*MAD2L1*		*DPM1*		*PPP1R18*
*ATF3*		*REEP5*		*EIF4B*		*BLZF1*		*IKBKB*
*NR4A1*		*CHMP5*		*RPL9*		*OLA1*		*GID4*
*SOCS3*		*SPCS1*		*SF3B1*		*SERINC1*		*MROH1*
*HNRNPL*		*VAMP3*		*NOP58*		*GMFB*		*POLR2I*
*BRWD3*		*DECR1*		*HNRNPDL*		*CUL5*		*FERMT3*
*LDLR*		*IDH1*		*NCL*		*SEL1L*		*TFEB*
*H2AFX*		*RAN*		*RPS25*		*CAPZA2*		*RPS6KA1*
*SOCS1*		*CASP1*		*CALM1*		*LAMTOR3*		*MKKS*
*TNFAIP3*		*UQCRQ*		*MAGOH*		*SH3BGRL*		*SLC35C1*
*GOLGB1*		*RAB10*		*EIF4A2*		*AGPS*		*ING1*
*AP4B1*		*MMADHC*		*RPS12*		*GNG2*		*ARHGAP1*
*OGT*		*COMMD8*		*RPS6*		*KIAA1033*		*PSMD4*
*DDX3X*		*LAMP2*		*TAF9B*		*NRAS*		*PHC2*
*POLR1C*		*PSMD14*		*HINT1*		*SPTLC1*		*S1PR4*
*KLHL11*		*PRDX3*		*BTAF1*		*SH3GLB1*		*MRPL15*
*BCL6*		*ACTR2*		*RPL7*		*DUSP3*		*ERAL1*
*ANAPC1*		*TNFSF13B*		*RPA3*		*TIMM10B*		*TMEM101*
*SF1*		*ARPC3*		*SOS1*		*C9orf72*		*LSM2*
*CXCR4*		*CTSS*		*RPL26*		*MAP3K1*		*MRPL40*
*RBMX*		*TGOLN2*		*BCL10*		*GFM1*		*ZNHIT2*
*ICAM1*		*MRPL13*		*RPS20*		*RHEB*		*RNF8*
*DDX24*		*SEC61G*		*RPL24*		*WRB*		*DOLK*
*MATR3*		*CETN2*		*MRPL32*		*EFR3A*		*SHMT2*
*RLIM*				*CSNK1G3*				

### MTRs Analysis

**Figure 6:**
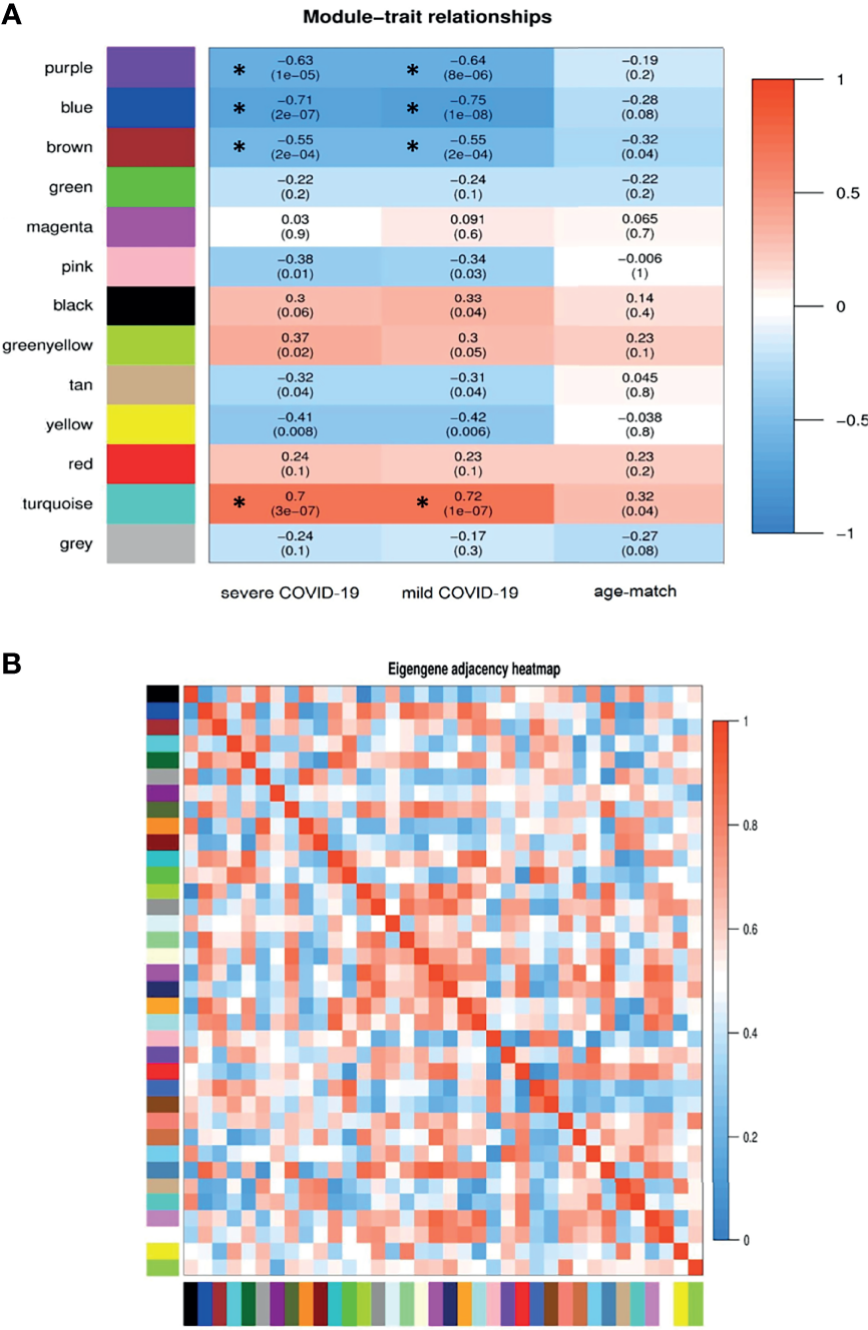
Module-trait relationships analysis. **(A)** Module-trait relationships (MTRs) between detected modules and disease severity of COVID-19. Module-trait relationships MTRs are obtained by calculating the correlation between the traits and the module eigengenes. The red and blue colors indicate strong positive correlation and strong negative correlation, respectively. Rows represent module eigengene (ME) and columns indicate disease severity of COVID-19. Asterisks corresponds significant highly-correlated values. **(B)** Eigengene adjacency heatmap indicate relationship among all the modules.

The weighted adjacency matrix was constructed at β = 10 whose scale-free topology fitting index (R2) was ≥ 0.80. After network construction, 12 co-expression modules (excluding grey module with 690 uncorrelated genes) were identified through hierarchical clustering and dynamic hybrid tree cutting with an average size of 784 genes. Disease severity related to COVID-19 that were used in MTRs included clinical signs measurements of COVID-19 such as flu-like illness (FLI), mild COVID-19, severe COVID-19. Among the significant modules: purple (R = −0.63, P = 1e−05), blue (R = −0.71, P = 2e−07), brown (R = −0.55, P = 2e−04), and turquoise (R = 0.7, P = 3e−07) modules were significant highly-correlated and yellow (R = −0.41, P = 0.008), tan (R = −0.32, P = 0.04), greenyellow (R = 0.37, P = 0.02), and pink (R = −0.38, P = 0.01) modules were significant moderately-correlated with severe COVID-19, respectively (**Figure 6A**). Also, purple (R = −0.64, P = 8e−06), blue (R = −0.75, P = 1e−08), brown (R = −0.55, P = 2e−04), and turquoise (R = 0.72, P = 1e−07) modules were significant highly-correlated and yellow (R = −0.42, P = 0.006), tan (R = −0.31, P = 0.04), black (R = 0.33, P = 0.04), and pink (R = −0.34, P = 0.03) modules were significant moderately-correlated with mild COVID-19, respectively (**Figure 6B**). Then, the significant highly-correlated modules were selected for downstream analysis. Briefly, the turquoise, blue, brown, and purple modules with module sizes of 2592, 1691, 1214 and 141 genes, respectively, were identified as significant highly-correlated modules with disease severity (**Figure 6A**). All of the detected modules were also identified with MP method. It is noteworthy that both WGCNA methods showed a similar ability to identify candidate modules during COVID-19, confirming each other results.

### Functional Enrichment Analysis of Highly-Correlated Modules

**Figure 7:**
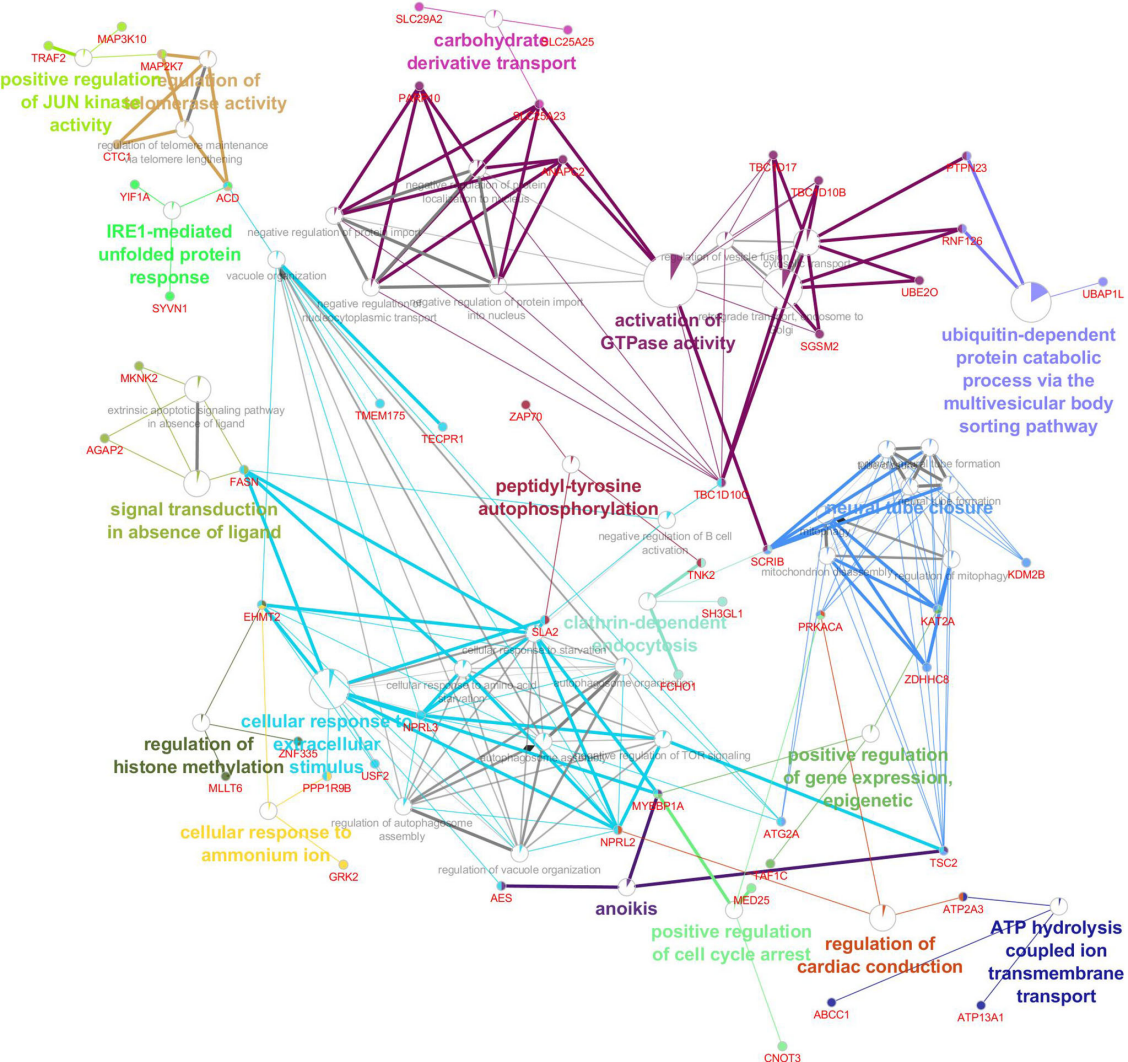
The Gene, Gene Ontology and pathway, related modules involved in the disease severity of COVID-19.

In order to understand the biological performance of significant highly-correlated modules with disease severity of COVID-19, functional enrichment analysis was performed and a total of 342 biological process and 93 KEGG pathways were significantly enriched in the respective modules. The turquoise module had the highest number of enriched terms and pathways, including 295 biological processes and 82 KEGG pathways. The most significant GO term and KEGG pathway in the turquoise module were “mitochondrial translational elongation (GO:0070125)”, “translational termination (GO:0006415)”, and “negative regulation of type I interferon-mediated signaling pathway (GO:0060339)”. On the other hand, 13 biological processes and 8 KEGG pathways were significantly enriched in the purple module. The most significant GO term and KEGG pathway in the purple module were “Fc gamma R-mediated phagocytosis” (GO:0006968, Adjusted P value = 2.27E-09) and “Autophagy” (Adjusted P value = 2.09E-06), respectively. Based on the functional enrichment analysis, among the significant highly-correlated modules with disease severity of COVID-19, blue, brown, lightgreen, grey60, lightyellow, pink, midnightblue, purple, and turquoise were associated with COVID-19 mechanisms. The identified modules in the COVID-19 samples with different colors as a heatmap and the relationship between them are shown presented in **Figure 6B**. **Figure 7** was constructed by ClueGO plugin and shows GO (describes our knowledge of the biological domain with respect to three aspects: Molecular Function, Cellular Component, and Biological Process (**Supplementary Table 10** **)**.

## Discussion

COVID-19, a global pandemic caused by SARS-CoV-2, has caused severe pulmonary conditions and many deaths (65–67). The prevalence of SARS-CoV-2 infection is rapidly increasing, and despite several vaccines, no cure is available (68–70). Development of potential therapies requires an understanding of molecular mechanisms of the disease and patient-pathogen interactions (6, 7). In this study, bioinformatics and systems biology approaches were used to elucidate molecular regulatory mechanisms responsible for COVID-19. Since the main aim of the study were to find key and hub high-traffic genes affecting the disease at the systems level, we used the Module Preservation approach. As well, all the modules and genes identified by the Module-trait Relationships approach were covered by the main study approach. Although the second approach requires a sufficient number of samples for each attribute, it can also be used as a validation of the MP method. It should also be noted that more studies are needed to increase the accuracy of the MTR analysis. As well, the main purpose of the study is to systematically study the COVID-19 in terms of the static aspect of biological processes. Based on differential co-expression network analyses, among the 21 modules identified in healthy controls, network connectivity patterns of 15 modules (72%) changed due to SARS-CoV-2 infection. That 15 of 21 modules were affected indicated the ability of SARS-CoV-2 to cause systemic disturbances in gene networks of healthy individuals. Investigating the function of these non-preserved modules should provide insights into molecular mechanisms and host-pathogen interactions, as well as identifying key genes, representing an important step for development of effective treatments. Moreover, in functional enrichment analyses, among the 15 non-preserved modules between healthy and COVID-19 samples, 9 modules including blue, brown, grey60, lightgreen, lightyellow, midnightblue, pink, purple, and turquoise had direct biological relationships with host immune responses and COVID-19 development.

### Blue Module

Based on GO and KEGG pathway analyses, the blue module was clearly closely related to SARS-CoV-2 pathogenesis. Co-expressed genes of this module were significantly enriched in KEGG pathways related to host inflammatory responses, including: Toll-like receptor signaling pathway; C-type lectin receptor signaling pathway; MAPK signaling pathway; NF-kappa B signaling pathway; and IL-17 signaling pathway.

Toll-like receptors (TLRs) are the most important pattern recognition receptors (PRRs); they recognize pathogen-associated molecular patterns (PAMPs), activate the innate immune system and regulate secretion of proinflammatory cytokines and the host inflammatory response (71, 72). Activation of the Toll-like receptor signaling pathway during SARS-CoV-2 infection leads to activation of a cascade of downstream pathways, thereby activating the MAPK and NF-kappa B signaling pathways, increasing production of type I interferons and proinflammatory cytokines such as *IL-1β*, *IL6*, and *TNF-α* (73–77). C-type lectin receptors, another type of PRRs, are involved in the proinflammatory response; their role has been investigated during Middle East respiratory syndrome coronavirus (MERS-CoV) and SARS-CoV-2 infections (78, 79). Moreover, SARS-CoV-2 ORF8 increased the expression level of proinflammatory cytokines by activating the IL-17 signaling pathway (80). Additionally, in the blue module, the biological processes associated with the cytokines included: positive regulation of cytokine production (GO:0001819); cellular response to cytokine stimulus (GO:0071345); cytokine-mediated signaling pathway (GO:0019221); inflammatory response (GO:0006954); and positive regulation of T cell cytokine production (GO:0002726). The vast cytokine release from the immune system in response to SARS-CoV-2 infection often exacerbates the proinflammatory response, with a cytokine storm in the lung that causes acute respiratory distress syndrome (ARDS), acute lung injury (ALI), decreased lung function, and host death (81, 82). Therefore, development of effective strategies for controlling or preventing excessive inflammation through these key pathways in the blue module, which regulate cytokine production, could enhance survival of COVID-19 patients (80, 83–85).

In the blue module, other important KEGG pathways and biological processes associated with the immune system included: TNF signaling pathway; Apoptosis; p53 signaling pathway; Influenza A; HIF-1 signaling pathway; Viral protein interaction with cytokine and cytokine receptor; and positive regulation of programmed cell death (GO:0043068).

The TNF signaling pathway is another key pathway responsible for the cytokine storm and lung injury during SARS-CoV-2 infection. Blocking the *NFkB1* inhibitory protein (IkB) induces the *NFkB1* transcription factor, which increases transcription of cytokine genes (86–88). This pathway has been considered an attractive therapeutic target for COVID-19-induced lung injury (86, 89).

The SARS-CoV-2 and MERS-Cov infections reduce percentages of peripheral blood mononuclear cells (PBMCs), e.g., T cells, by inducing apoptosis and contributing to disease pathogenesis (90, 91). In addition, induction of apoptosis in PBMCs during COVID-19 is more marked in severe clinical cases (90, 92). Moreover, severe COVID-19 is associated with numerous changes in PBMCs, e.g., lymphopenia (93) and lymphocyte apoptosis (especially T cells), with lymphopenia associated with severe COVID-19 (94, 95). The SARS-CoV-2-induced cytokine storm can exacerbate apoptosis in T cells, reducing their numbers in COVID-19 patients (96). More research is needed to mitigate lymphocyte apoptosis and lymphopenia in COVID-19 patients (94). Furthermore, in agreement with our research, in a transcriptomic study, in addition to apoptosis, the p53 signaling pathway was highly enriched in PBMCs of the COVID-19 group and may be associated with lymphopenia in COVID-19 patients (97).

The exacerbated inflammatory response due to severe/critical COVID-19 leads to ARDS, which can lead to multi-organ failure (MOF) and death (98). This imbalanced inflammatory response (cytokine storm) increases disease severity and is associated with high concentrations of circulating proinflammatory cytokines, e.g., *IL6*, *TNF*, and *IL1B* in lung tissue (93, 99–102). Interestingly, in this study, *IL6*, *TNF*, and *IL1B* were key hub-high traffic genes in the blue module. Interleukin-6 (*IL6*), a proinflammatory cytokine that enables virus to enter host cells and proliferate, is an important prognostic biomarker and a powerful predictor of COVID-19 mortality (103, 104); it is significantly increased in ICU patients, and was highly significantly correlated with COVID-19 mortality (105–107). Additionally, in an extensive proteomic study, *IL6* was an important candidate associated with disease severity (108). Given the key role of *IL6* in SARS-CoV-2 pathogenesis, blocking *IL6* signaling may prevent hyperinflammation and increase survival in COVID-19 patients (109–111). For example, tocilizumab (a monoclonal antibody against *IL6*) and convalescent plasma therapy (CPT) reduced *IL6* concentrations, controlled and relieved inflammation, and managed the cytokine storm in COVID-19 patients (112–115). Moreover, clinical trials are underway to investigate effects of *IL6* receptor and *IL1B* signaling blockade on COVID-19 treatment (100).

Tumor necrosis factor (*TNF*) is another key proinflammatory cytokine involved in COVID-19 hyperinflammation, as COVID-19 patients have increased *TNF* concentrations (116). *TNF* has a special nature and biological features that make it a promising target for treatment of COVID-19 (117). Anti-TNF therapies can manage inflammation in many human inflammatory diseases, e.g., chronic kidney disease (CKD), rheumatoid arthritis (RA) (118, 119), Crohn’s disease (120), psoriasis, psoriatic arthritis (121), and sepsis (122). There is ample evidence that anti-TNF therapies, in addition to reducing *TNF* concentrations and preventing inflammation, have additional therapeutic effects, including: suppressing formation of new blood vessels by modulating angiogenic vascular endothelial growth factor (*VEGF*) in patients with RA (123); reducing synovial expression of chemokines such as interleukin-8 (*IL8*) and monocyte chemotactic protein-1 (*MCP-1*) in RA patients (124); downregulating production of interleukin-18 (*IL18*) in RA patients (125); and reducing expression of adhesion molecules (126). Anti-TNF therapies reduce secretion of proinflammatory cytokines to normal (*IL1β*) or sub-normal (*IL6* and *IFN-γ*) in RA patients (127). Therefore, anti-TNF therapies may reduce many pathogenic proinflammatory cytokines during SARS-CoV-2 infection and be an effective immunomodulatory approach for treatment of COVID-19 patients (128, 129). In agreement with this study, these findings indicated the importance of *IL6*, *TNF* and *IL1B* hub-high traffic genes, considered potential targets (individually or in combination) for development of effective therapeutic immunomodulation strategies to manage COVID-19 hyperinflammation.

Identified hub-high traffic genes in the blue module included several important transcriptional regulatory factors, i.e., *NFKB1*and *HIF1A*. The nuclear factor-kappa b subunit-1 (*NFKB1*), an important transcription factor belonging to the NF-κB family, stimulates transcription of proinflammatory cytokines such as *IL6*, *TNF‐α*, and *IL1* as well as chemokines such as *IL8* (*CXCL8*), all with major roles in causing a COVID-19-induced cytokine storm (86, 87, 130, 131). The inflammation regulator *NFKB1* was significantly upregulated in response to SARS-CoV-2 infection; transcriptomic studies revealed its key role in regulation of differentially expressed genes (DEGs) between healthy individuals and patients with mild or severe COVID-19 (7, 10, 132–136). Furthermore, by targeting *NFKB1*, miR-9 regulates inflammatory pathways related to COVID-19 pathogenesis (137). Moreover, by binding to *NFKB1*, miR-27a-3p suppressed NF-κB activation and reduced acute lung injury in an animal model (138). These results demonstrated the vital role of the *NFKB1* hub-high traffic TF in the inflammatory response during COVID-19, consistent with targeting *NFKB1* as a potential treatment strategy for severe COVID-19 cases (85, 139). Fluoxetine, tiotropium, and andrographolide targeted *NFKB1*, suppressed inflammation, and reduced the cytokine storm in COVID-19 patients (86, 140, 141). Additionally, vitamin D blocked the TNF-induced *NFkB1* signaling pathway and blocked the cytokine storm in severe COVID-19 infections.

In severe cases of COVID-19, a hypoxic microenvironment activates Hypoxia Inducible Factor 1 Alpha (*HIF1A*), a master regulator that activates, recruits, and stabilizes immune cells such as macrophages and neutrophils at the site of inflammation; furthermore, these cells secrete inflammatory cytokines, causing a cytokine storm (142). Activation of *HIF1A* upregulates *VEGF*, which increases vascular leakage and destroys alveolar-interstitial-endothelial epithelial complex barriers (142). Conversely, activation of *HIF1A*, induces autophagy, a pathway through which SARS-CoV-2 increases its proliferation and progression in host cells (142, 143). Moreover, in agreement with our results, in a transcriptomic study, *HIF1A* had the highest connections in the GO, KEGG, and PPI networks of various clinical stages of COVID-19 (33). Thus, *HIF1A* inhibitors may interfere with processes that promote COVID-19 pathogenesis (144, 145).

Other critical hub-high traffic genes in the blue module included *EP300* (146, 147), *CDK1* (4, 148), *VEGFA* (149–151), *CTNNB1* (151, 152), *IL10* (9, 153–155), *SOCS1*, *SOCS3* (156), *SIRT1* (157, 158), *TFRC* (159–161), *HSP90AB1* (162), *CYCS* (163), *EZR* (164), *TNFAIP3*, *ICAM1* (10), and *PIK3R1* (139) as well as TFs such as *ATF4* (165), *ATF3* (166, 167), and *BCL6* (152) which have important roles in pathogenesis of SARS-CoV-2, and some of which are potential therapeutic targets. For instance, *SOCS1*/*3* antagonists may be prophylactic or therapeutic against the COVID-19 pandemic (156). Moreover, a significant increase in expression of endothelial cell adhesion molecules e.g., intercellular adhesion molecule 1 (*ICAM1*) in patients with severe COVID-19, is associated with COVID-19 severity and may cause coagulation disorders (168). Additionally, increased expression of *ICAM1* in COVID-19 patients contributes to replication of SARS-CoV-2 and provides a favorable environment for its survival in humans (135). Therefore, *ICAM1* protein can be considered a key target for treatment of COVID-19 patients (169, 170).

### Brown Module

The brown module was significantly enriched in immune-related pathways, including “NOD-like receptor signaling pathway”, “Necroptosis”, “tumor necrosis factor-mediated signaling pathway (GO:0033209)”, “interferon-gamma-mediated signaling pathway (GO:0060333)”, “T cell receptor signaling pathway (GO:0050852)”, “neutrophil mediated immunity (GO:0002446)”, and “neutrophil activation involved in immune response (GO:0002283)”.

NOD-like receptors (NLRs) are a specialized group of cytoplasmic PRRs with an important role in pathogenesis of a variety of inflammatory human diseases by regulating nuclear factor–kappa B (*NF-κB*) signaling, proinflammatory cytokines such as *IL1B*, and cell death (171). Moreover, in recent COVID-19 studies, the NOD-like receptor signaling pathway was among active pathways in response to the SARS-CoV-2 infection, implying this pathway may be a central mediator of severe COVID-19 (172, 173). Interestingly, this pathway and some of its members were reported to be important targets for reducing the cytokine storm in patients with severe COVID-19 (174–176).

Necroptosis or inflammatory cell death is immunogenic cell death in response to viral infection; it involves various processes, including clearance of virus-infected cells, inflammation, metabolic abnormalities, and embryonic development (177, 178). SARS-CoV-2 induces necroptosis through some of its viral proteins (179). Despite the immunological role of necroptosis, this pathway also contributes to pathogenesis of COVID-19. For example, synergism of interferon-gamma and tumor necrosis factor-mediated signaling can perpetuate a COVID-19-induced cytokine storm by stimulating programmed cell death (apoptosis and necroptosis) and increasing mortality (180). Moreover, increased secretion of mature *IL1B* through the necroptosis pathway can exacerbate the inflammatory response during SARS-CoV-2 infection (181). In addition, SARS-CoV-2 internalization by platelets induced them to undergo necroptosis and apoptosis, which can contribute to thrombosis (182). Furthermore, there is a major role of necroptosis in lung damage of COVID-19 patients (181). Therefore, blocking inflammatory cell death such as necroptosis may benefit COVID-19 patients by limiting tissue inflammation/injury (180). In this regard, Necrostatin-1 (Nec-1), an inhibitor of necroptosis, protected against complications of COVID-19 (183).

T-cells, lymphocytes in the cell-mediated adaptive immune response, are involved in viral clearance and long-term antiviral immunity (184). However, SARS-CoV-2 counteracts T-cell activity through specific immune escape mechanisms, e.g., T-cell apoptosis (90) or direct interactions with T-cell activator molecules (185). Moreover, severe COVID-19 is associated with dysregulation of T-cells, causing lymphopenia and exhaustion of CD4^+^ and CD8^+^ T cells (186–188). Conversely, activation of the T cell receptor signaling pathway in response to candidate drug treatment in COVID-19 patients indicates a key role of T-cells for limiting of SARS-CoV-2 infection during recovery (189). Despite their critical immunological role, T-cells may contribute to the pathogenesis of SARS-CoV-2. For instance, the inflammatory response and activation of T cells (especially T-helper 17 cells) can synergistically exacerbate the disease and prolong SARS-CoV-2 infection (15). Treatment strategies to activate/block T-cells in COVID-19 patients have been reviewed (190).

A major contributor of SARS-CoV-2 pathogenesis in the lungs of COVID-19 patients is excessive activation of neutrophils (191). Formation of neutrophil extracellular traps (NETs) by SARS-CoV-2-activated neutrophils can exacerbate inflammation-associated lung damage and cause severe pulmonary fibrosis in COVID-19 patients (192, 193). Moreover, high levels of NETs caused lung epithelial cell death *in vitro*, implying increased risk of mortality in COVID-19 patients (194). Conversely, formation of NETs by neutrophils in response to SARS-CoV-2 infection caused rapid vascular occlusion, altered microcirculation (195), and induced thrombosis (196). Therefore, NET-targeting approaches such as the use of NET-inhibitory factor (nNIF), could be a novel strategy to reduce lung damage and thrombotic responses during COVID-19 disease (197).

We also identified several significant terms related to mitochondrial activity in the brown module. These terms included “Oxidative phosphorylation”, “mitochondrial translational elongation (GO:0070125)”, “mitochondrial translational termination (GO:0070126)”, “mitochondrial ATP synthesis coupled electron transport (GO:0042775)” and “mitochondrial respiratory chain complex I assembly (GO:0032981)”. Mitochondria have a major role in maintaining cellular immunity, homeostasis and cell survival (198). Interestingly, there is emerging evidence that SARS-CoV-2 highjacks mitochondria in immune cells, manipulates mitochondrial activity to its advantage, and provides favorable conditions for viral replication within the mitochondrial structure (199). After entering the cell through the angiotensin-converting enzyme-2 (ACE-2) host receptor, the SARS-CoV-2 sends its genomic RNAs toward the mitochondria and, after manipulating it, affects various processes, including stimulation of cytokine production, mitophagy, iron storage, and platelet coagulation (200). Furthermore, SARS-CoV-2 may use mitochondrial-derived bilayer vesicles to hide within the cell (201). Additionally, host mitochondrial manipulation by SARS-CoV-2 ORF-9b released mitochondrial DNA (mt-DNA) into the cytoplasm, which activated an mtDNA-induced inflammasome, thereby suppressing innate and adaptive immune responses (201). Moreover, SARS-CoV-2 targets the oxidative phosphorylation pathway, causing a massive increase in reactive oxygen species (ROS) which increases *TNF* and *IL1B*, followed by hyperinflammation (202–205). Another mechanism of mitochondrial dysfunction by SARS-CoV-2 is excessive ferritin (hyperferritinemia) in mitochondria, causing oxidative stress (206). Furthermore, mitochondrial oxidative stress can impair glucose tolerance of cells and cause complications in diabetic patients (200). Given the importance of mitochondria in the pathogenesis of COVID-19, a clear understanding of biological interactions between mitochondria and the virus would be important to develop anti-SARS-CoV-2 strategies.

Previous COVID-19 studies reported that hub-high traffic genes in the brown module, such as *RBX1* (207–210), *PSMA3* (211), *PSMA6* (212), *PTEN* (213), *VPS29* (214, 215), *SNRPE* (216), *PSMA4* (217), *TXN* (218), *PTGES3* (219, 220), *RHOA* (57, 221, 222), *CANX* (209), *B2M* (223), *GDI2* (57, 224, 225), *TXNL1* (226), *SUMO1* (147, 227, 228), *PSMC2* (229), *REEP5* (230), *DECR1* (108), *RAB10* (224), *PRDX3* (231, 232), *ACTR2* (215), *TNFSF13B* (192, 233, 234), and *ARPC3* (215), have a central role in host-SARS-COV-2 interactions. For example, ARDS and ALI in patients with severe COVID-19 were directly associated with increased expression of the *PTEN* and *RHOA* hub-high traffic genes, respectively (213, 222). Additionally, a recent multi-omics study suggested the *GDI2* hub-high traffic gene as a potential biomarker for segregating COVID-19 positive cases (225). Moreover, SARS-CoV-2 disrupts PPAR-γ activity by suppressing *SUMO1*, resulting in a hyperinflammatory response in severe COVID-19 patients (147).

We identified the caspase-1 (*CASP1*) hub-high traffic gene in the brown module; it has a key role in the pathogenesis of SARS-CoV-2 and is associated with the severity of COVID-19 (235). Significant increases in the *CASP1* expression in patients with acute COVID-19 were reported in previous transcriptomic studies (236, 237). Indeed, activation of *CASP1* by activating inflammasomes in response to the SARS-CoV-2 infection activates *IL1B* and *IL18* and contributes to hypercytokinemia in COVID-19 patients (238–240). Besides, activation of *CASP1* promotes pyroptosis (a highly inflammatory and Caspase-1-dependent form of programmed cell death), which can lead to secretion of a wide range of inflammatory mediators (241, 242). Melatonin suppressed the lung cytokine storm in COVID-19 patients by reducing *CASP1* expression (243).

### Grey60 Module

Significant functional terms such as “B cell receptor signaling pathway”, “B cell activation (GO:0042113)”, and “regulation of B cell proliferation (GO:0030888)” demonstrated that the grey60 module was closely related to the humoral adaptive immunity. In agreement with our results, similar transcriptomic and systemic studies of COVID-19 reported enrichment of B cell receptor signaling pathway, B cell activation, and B cell proliferation pathways during SARS-CoV-2 infection (7, 244–246). Furthermore, B cell depleted patients infected with SARS-CoV-2 had prolonged disease (247). Remarkably, in 1 study, IgM memory B cells were commonly depleted in COVID-19 patients, which increased mortality (248). Moreover, given the relationship between B cell subset frequencies and clinical/laboratory parameters, these cells may be potential biomarkers for predicting the clinical outcome of COVID-19 (249). These findings supported the special importance of B cells and cell pathways during the humoral immune response in COVID-19 patients. Interestingly, we identified several hub-high traffic genes involved in regulating activity/development of B cells such as *CD19* (250), *IGLL5* (251, 252), *CD79B* (253), *CD79A* (254), *CD22* (255), *PNOC* (256), and *POU2AF1* (257), with a central role in information transfer within the grey60 module during humoral immunity in response to SARS-CoV-2 infection.

### Lightgreen Module

Investigation of KEGG pathways and biological processes of the lightgreen module revealed that co-regulated genes of this module were highly enriched in pathways such as “Th17 cell differentiation” and “Antigen processing and presentation”, as well as biological process such as “regulation of macrophage cytokine production (GO:0010935)”. As discussed in the “brown module” section, T cells can have dual actions; despite a key role in the immune response, they may also contribute to disease pathogenesis. Release of a wide range of cytokines (especially *IL6*) in response to SARS-CoV-2 infection enhanced differentiation of naive T cells into the T-helper 17 (TH17) phenotype (258–261). TH17 cells have a major role in inducing the proinflammatory effects of cytokines (*G-CSF*, *IL1B*, *IL6*, and *TNF*) and chemokines (*KC*, *MIP2A*, *IL8*, *IP10*, *MIP3A*) through secretion of inerleukin-17 (*IL17*), thus contributing to the cytokine storm and subsequent ARDS in COVID-19 patients (262–264). Moreover, an increase in the percentage of TH17 cells in response to the SARS-CoV-2 infection caused pregnancy complications, e.g., miscarriage, preterm labor, and preeclampsia in pregnant women with COVID-19 (265). Conversely, aberrant Th17 cell differentiation in COVID-19 patients increased the risk of autoimmune disorders, e.g., Guillain-Barré syndrome, multiple sclerosis, and rheumatoid arthritis (173, 266). Modulating compounds aimed at reducing the percentage of TH17 cells may suppress inflammation in COVID-19 patients (267).

Additionally, some lightgreen module hub-high traffic genes, including *GLUL* (268), *ACSL1* (269), *TRIB1* (236), *TUBA1B* (270), *PLEK* (271, 272), *HSPA5* (273, 274), and *IRAK3* (275) may contribute to the pathogenesis of COVID-19. For example, SARS-CoV-2 spikes can drive the infection process of host cells by binding to cell-surface heat shock protein A5 (*HSPA5*) receptor (273, 276). However, targeting this receptor using natural compounds, e.g., phytoestrogens or estrogens, can prevent SARS-CoV-2 from binding to stressed cells and thus limit infection (277). Conversely, the *IRAK3* hub-high traffic gene is an important inflammatory mediator associated with asthma susceptibility (278, 279) and SARS-CoV-2 increases risk of asthma in COVID-19 patients by increasing expression of this gene (275). Additionally, a network-based study recently suggested the *IRAK3* hub-high traffic gene as a potential therapeutic target against COVID-19 (280). Moreover, we identified aryl hydrocarbon receptor (*AHR*) hub-high traffic TF among the central genes of the lightgreen module. Increased expression of this transcriptional regulatory factor in response to the SARS-CoV-2 infection interfered with antiviral immunity in the host, as *AHR* suppressed production of type I interferons in the COVID-19 patients and increased SARS-CoV-2 replication in host cells (281). Therefore, pharmacologic *AHR* blockade approaches may be effective to enhance host antiviral immunity and reduce viral replication in COVID-19 patients (281).

### Lightyellow Module

Functional examination of the lightyellow module suggested that this module was only enriched in the “Natural killer cell mediated cytotoxicity” KEGG pathway. Additionally, some important hub-high traffic genes involved in the cytotoxic activity of natural killer cells during COVID-19 were *NCAM1* (252, 282), *GZMA*, *NKG7* (283, 284), *KLRD1* (285, 286), *GZMB* (150, 287, 288), and *PRF1* (289–291). Natural killer (NK) cells are key innate immune cells with a central role in killing virus-infected cells (292). Decreased NK cells during SARS and SARS-CoV-2 infections, followed by an increase during the recovery period indicates an inverse relationship between number of NK cells and disease severity (188, 293, 294), with a decrease in their number early in the disease process promoting progression of coronavirus infection (294).

Among the hub-high traffic genes in the lightyellow module, *GZMB* and *PRF1* had the most important role in NK cells cytotoxicity. Perforin glycoprotein, expressed by the *PRF1* gene, has an important role in the cytolytic activity of cytotoxic T cells and NK cells and causes pores in the membrane of target cells (295, 296). Granzyme B is a key serine protease expressed in cytotoxic T cells and NK cells by the *GZMB* gene; it enters the target cells through membrane pores created by perforin and induces apoptosis, eliminating infected cells (297, 298). Interestingly, decreased expression of both *PRF1* and *GZMB* hub-high traffic genes in severe COVID-19 patients lead to dysfunction of NK cells cytotoxicity, indicating that both genes are essential for NK cells activity (155, 299). Conversely, genetic mutations in the *PRF1* gene impaired NK cells cytotoxicity, implicating this gene in the antiviral activity of NK cells (300–302). Given the importance of NK cells and their genes/proteins in antiviral activity, as well as their reduction in COVID-19 patients, development of treatment strategies to restore NK cells may control infection in the early stages of COVID-19 (303). Moreover, other genes in the lightyellow module, e.g., *ERBB2* (151) and *ADRB2* (275, 304), as well as *TBX21* (305–307) hub-high traffic TF, have critical roles during SARS-CoV-2 infection. For instance, in addition to the *IRAK3* hub-high traffic gene (described in the lightgreen module section), increased *ADRB2* hub-high traffic gene expression during SARS-CoV-2 infection also increased the risk of asthma in COVID-19 patients (275).

### Midnightblue Module

The only significant enriched term in midnightblue was “regulation of immune response (GO:0050776)”. In this module, we also identified several hub-high traffic genes that were crucial in regulating the host immune response during COVID-19. These hub-high traffic genes included *CDKN2A* (308, 309), *OASL* (282, 310), *CCL5* (311–316), *KIF2C* (4), and *CD8A* (317). Among them, the *CCL5* hub-high traffic gene has the most important role in regulating the inflammatory response and the host immune system; however, its excessive expression can amplify inflammatory responses toward immunopathology (e.g., cytokine storm) (311, 314). C-C motif ligand 5 (*CCL5*) also known as RANTES, is a chemotactic molecule in the CC family of inflammatory chemokines, expressed by virus-infected airway epithelial cells and macrophages, which promotes trafficking of proinflammatory leukocytes to the site of infection and stimulates cytokine production (314, 318–320). A massive increase in secretion of pro-inflammatory molecules, including chemokines such as *CCL5* and other cytokines in response to SARS-CoV-2 infection, cause a cytokine storm (84, 321–323). Multiple-fold increases in *CCL5* expression in severe COVID-19 patients compared to healthy individuals are well documented (311, 312, 315, 324). Additionally, there was a significant increase in *CCL5* expression in non-surviving COVID-19 patients compared to severe, mild and healthy groups (313). Furthermore, high levels of *CCL5* cause liver disorders and acute kidney injury (AKI) in COVID-19 patients (314, 318, 325). Therefore, early intervention to prevent overexpression of *CCL5* can restore immune balance in COVID-19 patients and prevent disease progression (311, 314). Moreover, 2′–5′‐oligoadenylate synthetase‐like (*OASL*), the other hub-high traffic gene of midnightblue module, is an interferon-stimulating gene with a key role in antiviral defense mechanisms (326) and with potential as a diagnostic biomarker for COVID-19 (327).

### Pink Module

In the pink module, genes were observed in functional enriched terms such as “Coronavirus disease”, “Ribosome”, “ribosome assembly (GO:0042255)”, “mitochondrial transport (GO:0006839)”, and “translation (GO:0006412)”. Ribosomes are complex molecular machines that synthesize proteins by translating mRNA into polypeptides (328). Interestingly, in the infection stage, nonstructural protein 1 (Nsp1) from SARS-CoV-2 bound to the host ribosome and hijacked it to (1) disrupt the mechanism of host cellular translation and protein production (2), inhibiting all cellular antiviral defense mechanisms (3), initiating translation of viral mRNAs and subsequently viral protein production, and (4) increasing viral replication efficiency (329–335). Therefore, inhibitory mechanisms to target ribosome-SARS-CoV-2 interactions may be used to treat COVID-19 (331). Additionally, some pink module hub-high traffic genes, such as *RPL9*, *RPS27A*, *RPL26*, *RPS12* (336), *RPS6* (228), *RPL5*, *RPL7* (337), *RPGR* (173), and *RPS20* (338) were highly enriched in the ribosome pathway and involved in SARS-CoV-2-ribosome interactions. For example, the increase in ribosomal protein L9 (*RPL9*) expression was attributed to SARS-CoV-2 hijacking the host translation machine (339).

We identified the heat shock protein 90 alpha family class A member 1 (*HSP90AA1*) hub-high traffic gene in the pink module, which has an important role in development of SARS-CoV-2 infection (340) and has a significant increase in expression in severe COVID-19 patients compared to the non-severe group (341, 342). Furthermore, this hub-high traffic gene is also involved in comorbidities associated with COVID-19, e.g., cardiovascular diseases (CVDs) and has been suggested as a potential therapeutic target for these complications (139, 343, 344). We also identified mitochondrial 60-kDa heat shock protein (*HSPD1*) hub-high traffic gene in the pink module. High concentrations of circulatory *HSPD1* in severe COVID-19 patients are associated with cardiac failure and are a potential clinical biomarker for heart disorders in COVID-19 patients (345).

Other hub-high traffic genes in this module, such as *BTAF1* (216), *EZH2* (346), *NPM1* (347), *TPT1* (151, 269, 348), *LDHA* (217, 268), *PTPRC* (189), *SOS1* (349, 350), *DDX5* (351, 352), *BCL10* (162, 353), *EEF1B2* (354), *CALM1* (344), *EIF4A2*, *EIF4B* (336, 355, 356), *EEF1A1* (269, 354), *HNRNPA1* (228, 347), and *IFNG* (8, 357, 358), have important roles in development/inhibition of COVID-19. Overexpression of SARS-CoV-2 ORF6 caused *HNRNPA1* nuclear accumulation, subverting the host mRNA export system and reducing nucleus size (359). Moreover, *IFNG* hub-high traffic gene neutralizers have been predicted as antagonists of COVID-19 biology (8, 357).

### Purple Module

The immune-related enriched pathways in the purple module included “Fc gamma R-mediated phagocytosis” and “Autophagy”; these 2 pathways were significantly enriched in previous COVID-19 studies (189, 360, 361). Autophagy is an evolutionarily conserved mechanism to selectively eliminate damaged organelles, misfolded proteins, and intracellular pathogens (362). Components are enclosed in an autophagosome, which fuses with a lysosome to form an autolysosome that breaks down its contents (363). However, there is growing evidence that coronaviruses, such as SARS-CoV-1, MERS-CoV, and SARS-CoV-2, manipulate/hijack the autophagy pathway to accelerate their replication and complete their life cycle (364–368). Accordingly, inhibitory mechanisms and blockade of the autophagy pathway may be a novel therapeutic strategy (369–373).

Additionally, some of the purple module hub-high traffic genes, such as *MAPK1* (189, 374), *CUL2* (210), *CMTM6* (162), *TXNRD1* (375), *RAB1A* (376), *DICER1* (377), *RAB5A* (209), *HSP90B1* (343), *MAGT1* (378), *ADAM10* (379, 380), *SNX2* (89), *OLA1* (381), *SPTLC1* (382), *SH3GLB1* (383), *TIMM10B* (384), and *CREB1* (385) hub-high traffic TF, which are central for information exchange in this module, are potential targets for development of COVID-19 therapeutic strategies. Among these, the mitogen-activated protein kinase 1 (*MAPK1*) hub-high traffic gene is a potential core target for many anti-COVID-19 therapeutic strategies (189, 374, 386–390). Perhaps targeting the *MAPK1* hub-high traffic gene with guanfacine or desipramine may be effective for treating COVID-19 associated comorbidities, such as diabetes, CVDs, and chronic kidney diseases (CKDs) (139). Interestingly, the ras-related protein Rab-5A (*RAB5A*) is involved in various autophagy processes such as autophagosomal maturation and early autolysosome formation, and the SARS-CoV-2 enhances autophagy by increasing expression of this gene to accelerate viral replication (391). Furthermore, *RAB5A* is a key host protein for interaction with SARS-CoV-2 and may be an important target for inhibiting COVID-19 progression (209).

### Turquoise Module

Based on the functional terms of the turquoise module such as “mitochondrial translational elongation (GO:0070125)”, “translational termination (GO:0006415)”, and “negative regulation of type I interferon-mediated signaling pathway (GO:0060339)”, this module may have an important role in pathogenesis and development of SARS-CoV-2. In agreement with our results, COVID-19 suppressed type I interferon signaling and impaired their responses (6, 392, 393). Additionally, some SARS-CoV-1, MERS-CoV, and SARS-CoV-2 proteins decreased production and impaired type I interferon signaling (394). Accordingly, disruption of the type I interferon signaling, key antiviral mediators, contributed to the pathogenesis of COVID-19 (395). Indeed, impaired type I interferon signaling and exacerbation of the inflammatory response are hallmarks of severe/critical COVID-19 (234, 396). Therefore, type I interferons may be an intervention for SARS-CoV-2 infection (397).

Additionally, in terms of individual genes in the turquoise module, we identified hub-high traffic genes such as *UTP14A* (398), *RUVBL2* (139), *PRKCD* (280, 399), *KEAP1* (400–402), *CEBPA* (TF) (403), *RPL26L1* (404), *PTPN6* (255), *PPARA* (TF) (405), *TNFRSF1A* (406–408), *IKBKB* (275, 409), *TFEB* (TF) (410, 411), *PSMD4* (412), and *ARHGAP1* (413), important components in immunopathogenesis of SARS-CoV-2. For example, the TNF receptor superfamily member 1A (*TNFRSF1A*) hub-high traffic gene may be a biomarker of mortality in severe COVID-19 patients, as increased expression of this gene was significantly correlated with mortalities (414). We also identified tumor protein p53 (*TP53*) hub-high traffic TF in the turquoise module, with an important role in the pathogenesis of COVID-19 (415). Moreover, in an animal model of COVID-19, ALI in the infected group was associated with an increase in *TP53* expression (416). Besides, 1 study reported that an increase in the expression of *TP53* led to the induction of apoptosis in PBMCs and thus reduced their frequencies in COVID-19 patients (97). Furthermore, this TF is associated with some human inflammatory diseases such as RA (417). In agreement with us, this TF was identified as a hub gene in several network-based COVID-19 studies (347, 418–421). Therefore, this TF may have an immunoregulatory role during SARS-CoV-2 infection and be a potential therapeutic target.

As well as, different proteomic databases were explored. More data bases were related to SARS-CoV-2 structure and different situations and tissues. Therefore COVID-19 Immune Atlas (https://covid19cellatlas.com/#/) was selected. Hospitalized COVID-19 patients and age-matched healthy controls were recruited. No differences in age, sex, body mass index (BMI), viremia, or time from symptom debut until hospital admission were present between moderate and severe patients. Based on different cells in the Atlas below results were achieved:

Both moderate and severe COVID-19 patients were characterized by the progressive expansion of CD16 neutrophils, which also expressed lower levels of CD177, CD11b, and CD62L and higher levels of CD66b and LOX-1, a phenotype compatible with neutrophil immaturity. Intriguingly, a positive correlation between type 1 inflammatory mediators (e.g., IFNγ and CXCL10) and eosinophil activation was observed, suggesting that, particularly in moderately affected patients, part of the granulocyte compartment could be actively participating in the efficient viral clearance, similarly to what can occur upon influenza infection.

CXCR4 is one of the most highly expressed basophil receptors in COVID-19 patients and might be implicated in basophil transendothelial migration. CD63 expression on basophils can be induced by cross-linking of CD62L and CD11b, among other stimuli. Therefore, the up-regulation of CD62L, CD63, CD11b, and CXCR4 on basophils observed during the acute phase of COVID-19 and their normalization after viral clearance might imply a role of this phenotype in COVID-19 pathophysiology.

We found increased levels of CXCR4 in both moderate and severe patients. Other recent studies have also observed increased levels of CXCR4 with levels higher in severe than in non-severe COVID-19 patients. We also observed a characteristic increase in ASC expansion during acute COVID-19 in both severe and moderate patients; however, no difference was observed between the two patient groups. Expansion of ASCs during viral infections has been shown to be a good predictor of the development of neutralising antibodies and B cell memory. Additionally, during viral infections, ASCs can produce large amounts of antibodies as long as viral shedding occurs, suggesting that ASCs play an active role in infection clearance. We found that the majority of patients had neutralizing antibody titres during the acute phase, perhaps originating from the expanded ASC population. Taken together, the observed ASC expansion during acute COVID-19 may play an important role in SARS-CoV-2 clearance and disease control. We highlight the strength of analyzing secretion of multiple cytokines at once, as this approach provides information on cytokine co-expression patterns and identification of polyfunctional T cells, which are superior in their cytokine secretion capacity and therefore may be important in antiviral defence upon re-exposure to SARS-CoV-2.

Innate lymphoid cells were largely depleted from the circulation of COVID-19 patients compared with healthy controls. Remaining circulating ILCs revealed decreased frequencies of ILC2 in severe COVID-19, with a concomitant decrease of ILC precursors (ILCp) in all patients, compared with controls. ILC2 and ILCp showed an activated phenotype with increased CD69 expression, whereas expression levels of the chemokine receptors CXCR4 and CCR4 were significantly altered in ILC2 and ILCp, and ILC1, respectively. The activated ILC profile of COVID-19 patients was associated with soluble inflammatory markers, while frequencies of ILC subsets were correlated with laboratory parameters that reflect the disease severity.

Unsupervised analysis of peripheral blood NK cells furthermore identified distinct NK cell immunotypes that were linked to disease severity. Hallmarks of these immunotypes were high expression of perforin, NKG2C, and Ksp37, reflecting increased presence of adaptive NK cells in circulation of patients with severe disease. Last, arming of CD56 NK cells was observed across COVID-19 disease states, driven by a defined protein-protein interaction network of inflammatory.

We found that T cell activation, characterized by expression of CD38, was a hallmark of acute COVID-19. Many of these T cells also expressed HLA-DR, Ki-67, and PD-1, indicating a combined activation/cycling phenotype, which correlated with early SARS-CoV-2-specific IgG levels and, to a lesser extent, plasma levels of various inflammatory markers. Our data also showed that many activated/cycling T cells in the acute phase were functionally replete and specific for SARS-CoV-2.

The present findings provided new knowledge regarding molecular mechanisms responsible for COVID-19 as well as host-virus interactions. The current study emphasized the ability of SARS-CoV-2 to cause systemic perturbation in host biological-immunologic gene networks and then manipulate them to accelerate viral replication and progression of COVID-19. Building on these findings, further research is needed to more fully characterize molecular fingerprints underlying SARS-CoV-2 infection for development of drugs and vaccines against COVID-19.

## Conclusion

COVID-19 is a global pandemic that has affected many and despite much effort, no effective treatment is yet available. A systems biology approach was used to investigate molecular regulatory mechanisms responsible for COVID-19 at the systemic level. It is noteworthy that both WGCNA methods showed a similar ability to identify candidate modules during COVID-19, confirming each other results. Differential co-expression network analysis revealed that the topological structure of 72% (15 of 21) of the modules were affected due to the SARS-CoV-2 infection. Moreover, based on functional enrichment analysis, among the 15 non-preserved modules between healthy controls and COVID-19 patients, 9 modules were directly involved in the host immune response and COVID-19 immunopathogenesis. Integration of co-expression networks based on the hub genes with PPI networks identified 290 hub-high traffic genes. Indeed, these genes have the highest degree of connection in co-expression networks and the highest BC score in co-expressed hub gene-based PPI networks and can be considered promising therapeutic targets for development of treatment strategies against COVID-19. Interestingly, most of these hub-high traffic genes had a central role in immunopathogenesis of COVID-19 and were directly related to disease severity and mortality. Future research is needed to validate hub-high traffic genes reported in this study, especially those whose role in the pathogenesis of SARS-CoV-2 is yet unclear.

## Data Availability Statement

RNA-seq raw reads of COVID-19 patients and healthy individuals were obtained from the Gene Expression Omnibus (GEO) database at the National Center for Biotechnology Information (NCBI) under the accession number of GSE152418.

## Author Contributions

AH and AB conceived the ideas. AH and AB designed the study. AH, AB, and NS analyzed the data. AH, AB, BA, BH, and FF interpreted the data. AH wrote the main manuscript. HG, GJ, BH, BA, and MR helped to write manuscript. AB, JK, and HB reviewed and edited the manuscript. All authors contributed to the article and approved the submitted version.

## Conflict of Interest

The authors declare that the research was conducted in the absence of any commercial or financial relationships that could be construed as a potential conflict of interest.

## Publisher’s Note

All claims expressed in this article are solely those of the authors and do not necessarily represent those of their affiliated organizations, or those of the publisher, the editors and the reviewers. Any product that may be evaluated in this article, or claim that may be made by its manufacturer, is not guaranteed or endorsed by the publisher.

## Abbreviations

AKI, acute kidney injury; ALI, acute lung injury; ARDS, acute respiratory distress syndrome; BC, betweenness centrality; COVID-19, corona virus disease 2019; CVD, cardiovascular disease; CPM, count per million; GO, gene ontology; KEGG, Kyoto encyclopedia of genes and genomes; MERS-CoV, middle east respiratory syndrome coronavirus; MM, module membership; MOF, multi organ failure; PBMC, peripheral blood mononuclear cell; PPI, protein-protein interaction; RA, rheumatoid arthritis; RNA-seq, RNA-sequencing; SARS-CoV-2, severe acute respiratory syndrome corona virus 2; TF, transcription factor; TOM, topological overlap matrix; WGCNA, weighted gene co-expression network analysis.
