# Corrigendum: Differential co-expression network analysis reveals key hub-high traffic genes as potential therapeutic targets for COVID-19 pandemic

**DOI:** 10.3389/fimmu.2025.1572273

**Published:** 2025-03-07

**Authors:** Aliakbar Hasankhani, Abolfazl Bahrami, Negin Sheybani, Behzad Aria, Behzad Hemati, Farhang Fatehi, Hamid Ghaem Maghami Farahani, Ghazaleh Javanmard, Mahsa Rezaee, John P. Kastelic, Herman W. Barkema

**Affiliations:** ^1^ Department of Animal Science, College of Agriculture and Natural Resources, University of Tehran, Karaj, Iran; ^2^ Nuclear Agriculture Research School, Nuclear Science and Technology Research Institute, Karaj, Iran; ^3^ Department of Animal and Poultry Science, College of Aburaihan, University of Tehran, Tehran, Iran; ^4^ Department of Physical Education and Sports Science, School of Psychology and Educational Sciences, Yazd University, Yazd, Iran; ^5^ Biotechnology Research Center, Karaj Branch, Islamic Azad University, Karaj, Iran; ^6^ Department of Medical Mycology, School of Medical Science, Tarbiat Modares University, Tehran, Iran; ^7^ Department of Production Animal Health, Faculty of Veterinary Medicine, University of Calgary, Calgary, AB, Canada

**Keywords:** systems biology, systems immunology, WGCNA, hub-high traffic genes, immunopathogenesis, therapeutic targets in infectious diseases, COVID-19 pandemic

In the published article, there was an error in affiliation 2. Instead of “Biomedical Center for Systems Biology Science Munich, Ludwig-Maximilians-University, Munich, Germany”, it should be “Nuclear Agriculture Research School, Nuclear Science and Technology Research Institute, Karaj, Iran”.

The authors apologize for this error and state that this does not change the scientific conclusions of the article in any way. The original article has been updated.

