# Supplementary material for: Differential Co-Expression Network Analysis Reveals Key Hub-High Traffic Genes as Potential Therapeutic Targets for COVID-19 Pandemic
Source: Front Immunol. 2021 Dec 15;12:789317. doi: 10.3389/fimmu.2021.789317 (PMC8714803; doi:10.3389/fimmu.2021.789317)
Supplement: Supplementary file 1 [file Presentation_1.pptx]

## Slide 1
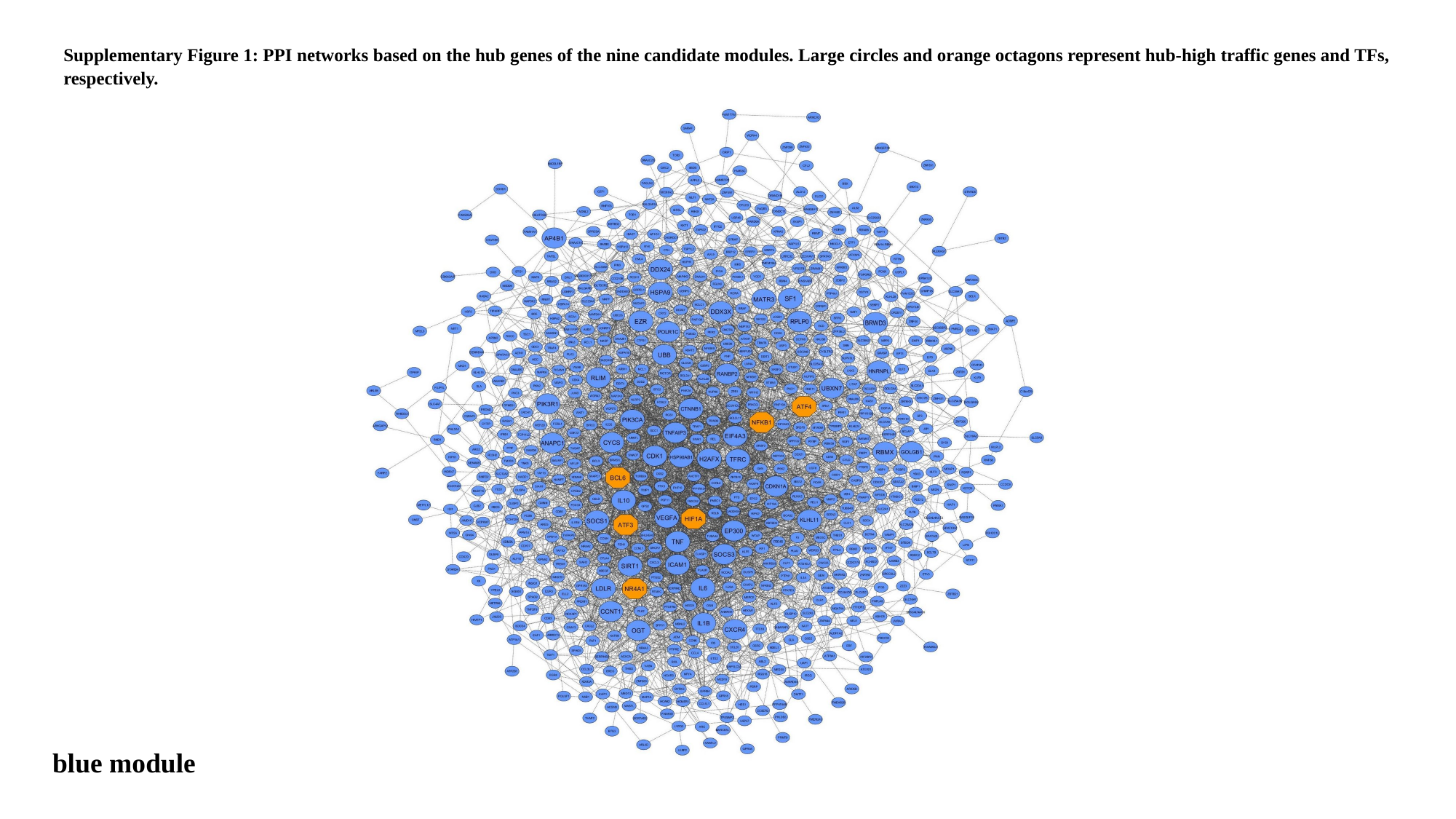

Supplementary Figure 1: PPI networks based on the hub genes of the nine candidate modules. Large circles and orange octagons represent hub-high traffic genes and TFs, respectively.
blue module

## Slide 2
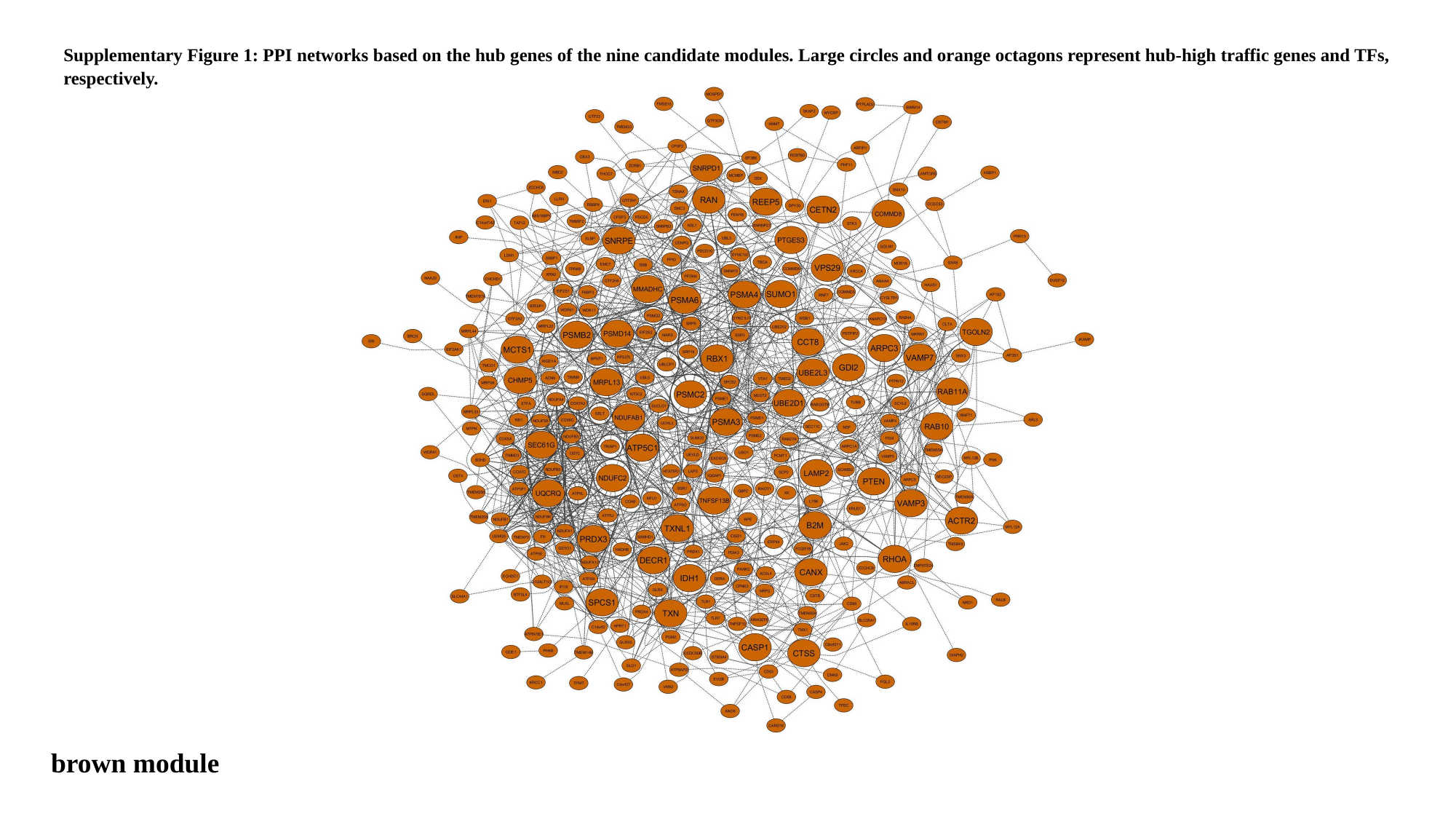

Supplementary Figure 1: PPI networks based on the hub genes of the nine candidate modules. Large circles and orange octagons represent hub-high traffic genes and TFs, respectively.
brown module

## Slide 3
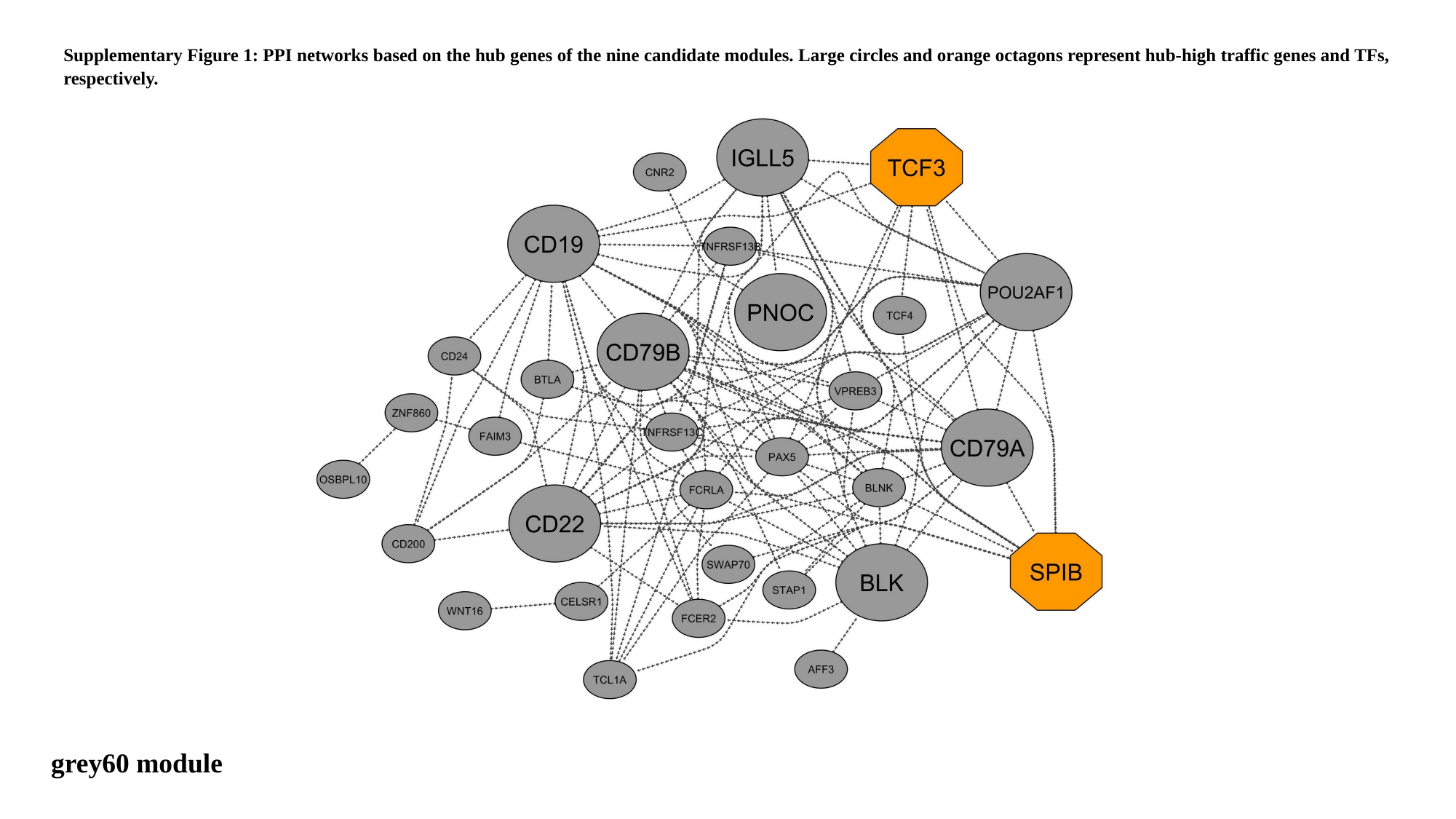

Supplementary Figure 1: PPI networks based on the hub genes of the nine candidate modules. Large circles and orange octagons represent hub-high traffic genes and TFs, respectively.
grey60 module

## Slide 4
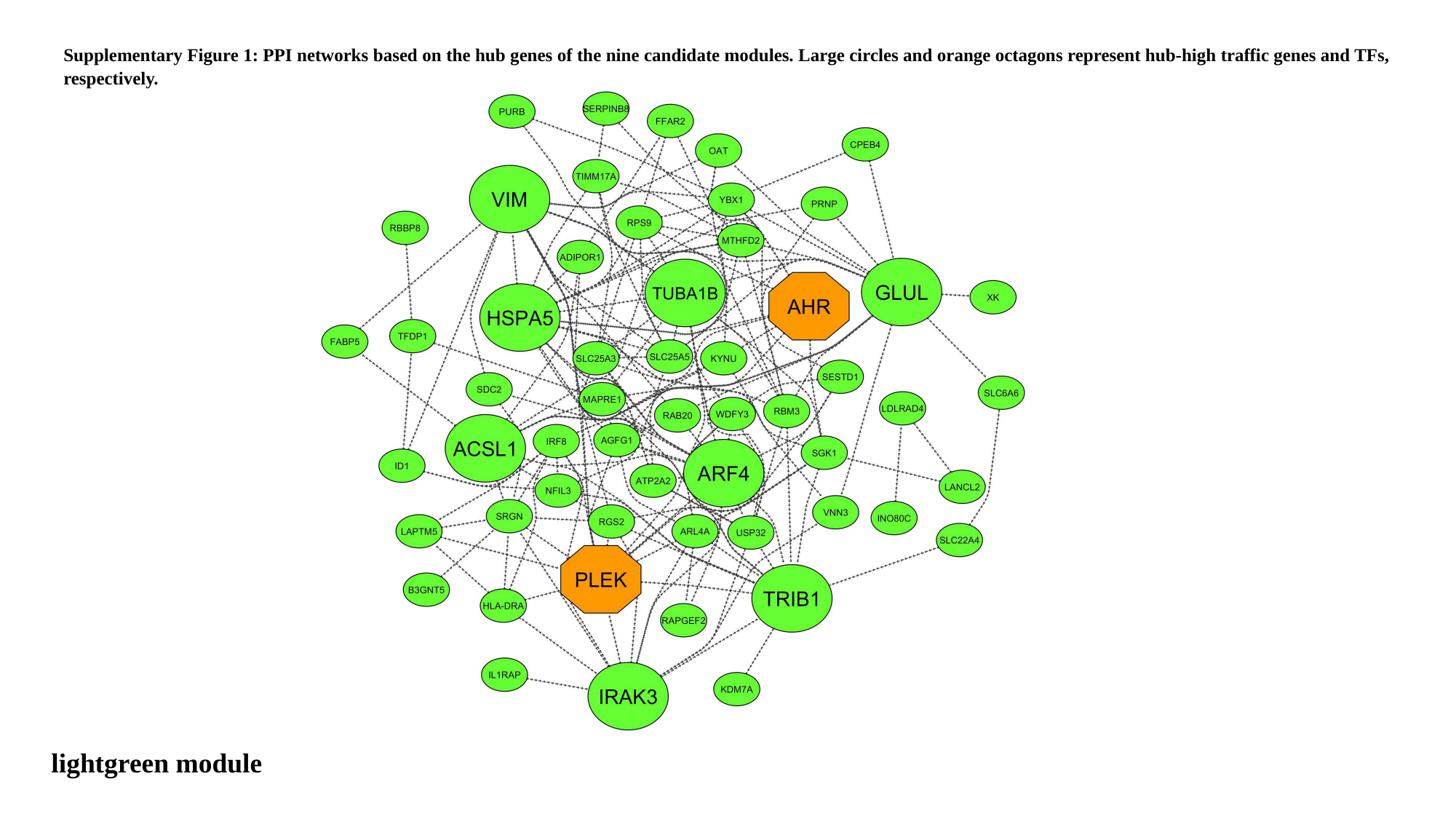

Supplementary Figure 1: PPI networks based on the hub genes of the nine candidate modules. Large circles and orange octagons represent hub-high traffic genes and TFs, respectively.
lightgreen module

## Slide 5
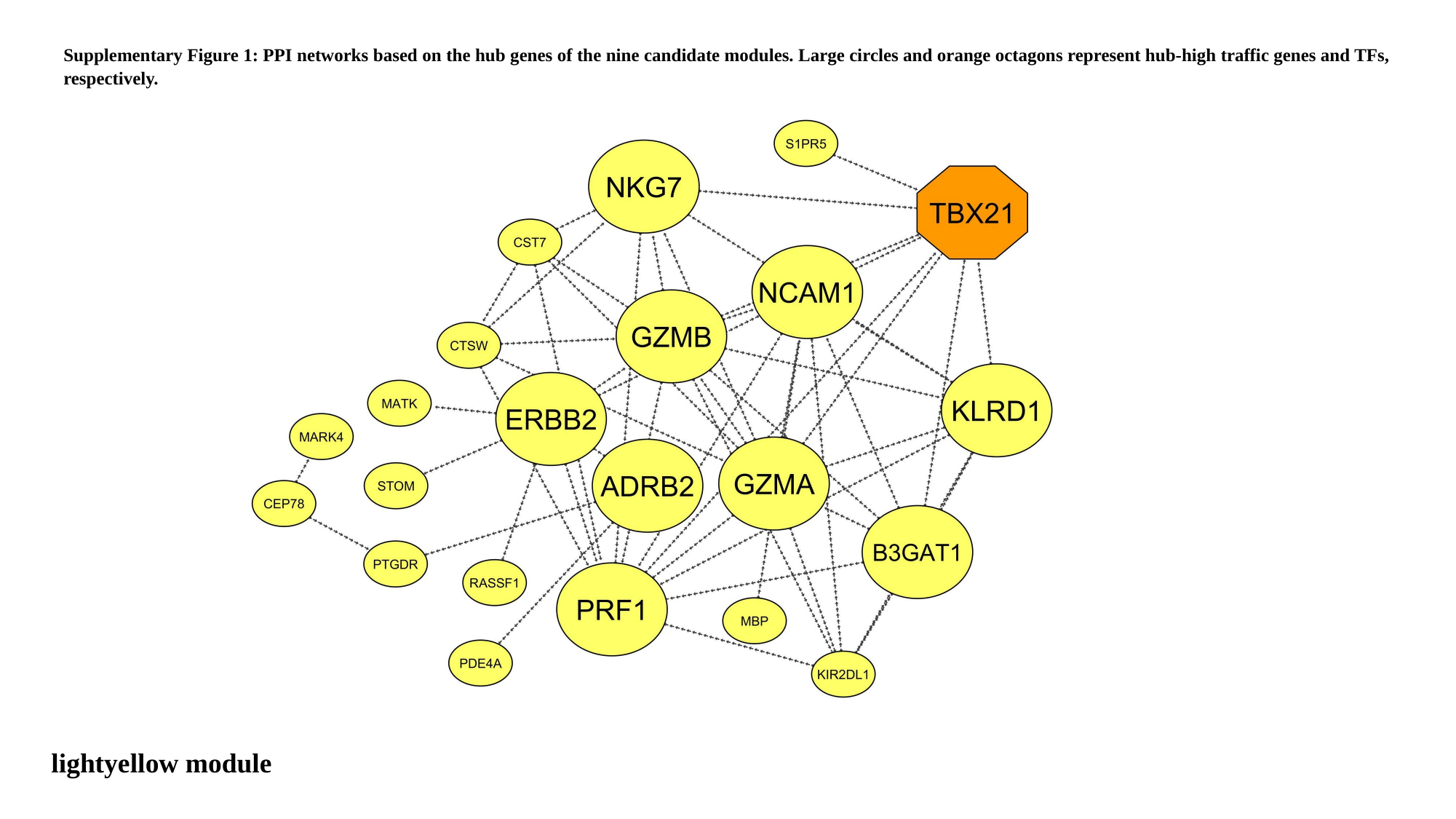

Supplementary Figure 1: PPI networks based on the hub genes of the nine candidate modules. Large circles and orange octagons represent hub-high traffic genes and TFs, respectively.
lightyellow module

## Slide 6
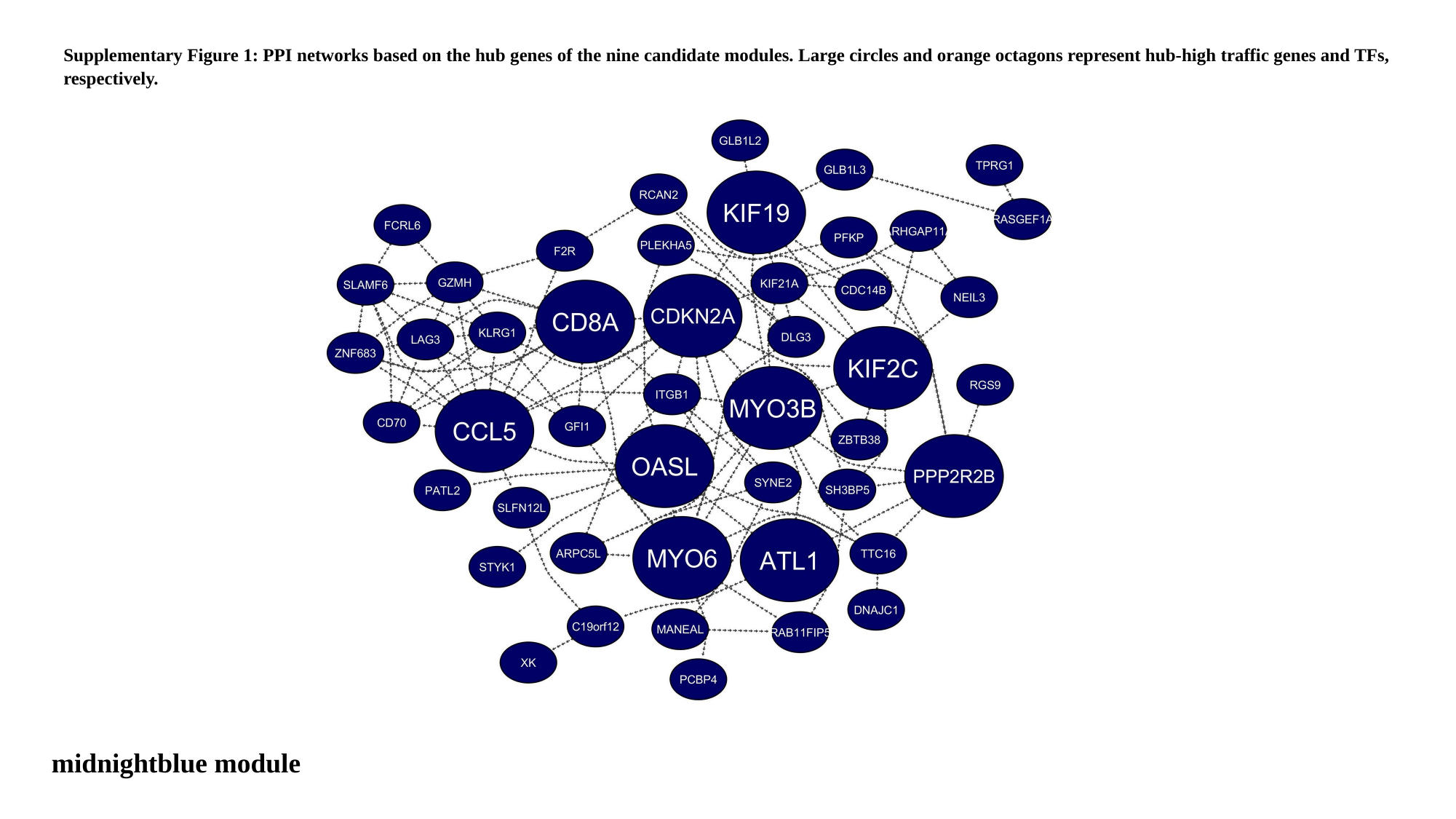

Supplementary Figure 1: PPI networks based on the hub genes of the nine candidate modules. Large circles and orange octagons represent hub-high traffic genes and TFs, respectively.
midnightblue module

## Slide 7
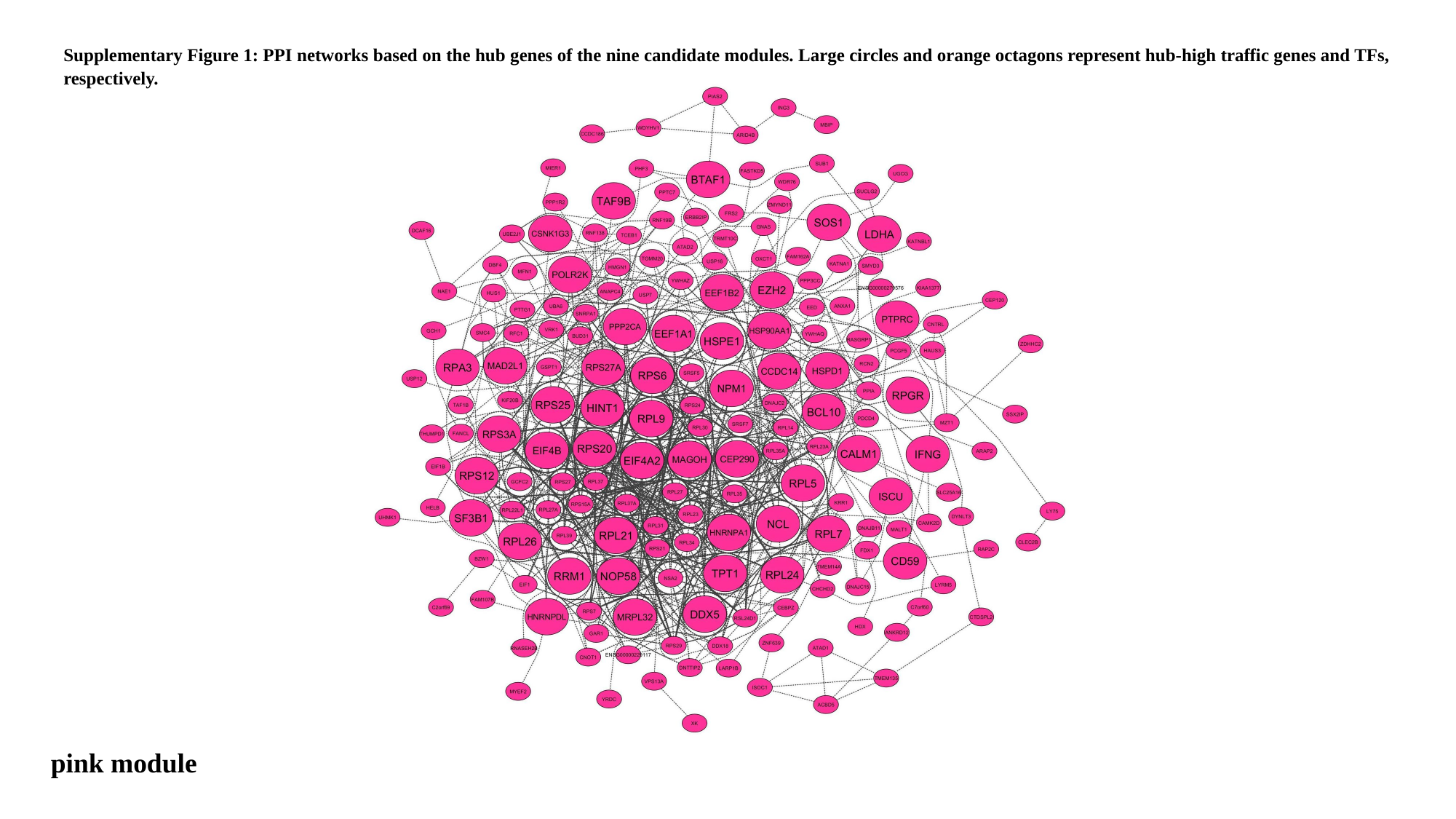

Supplementary Figure 1: PPI networks based on the hub genes of the nine candidate modules. Large circles and orange octagons represent hub-high traffic genes and TFs, respectively.
pink module

## Slide 8
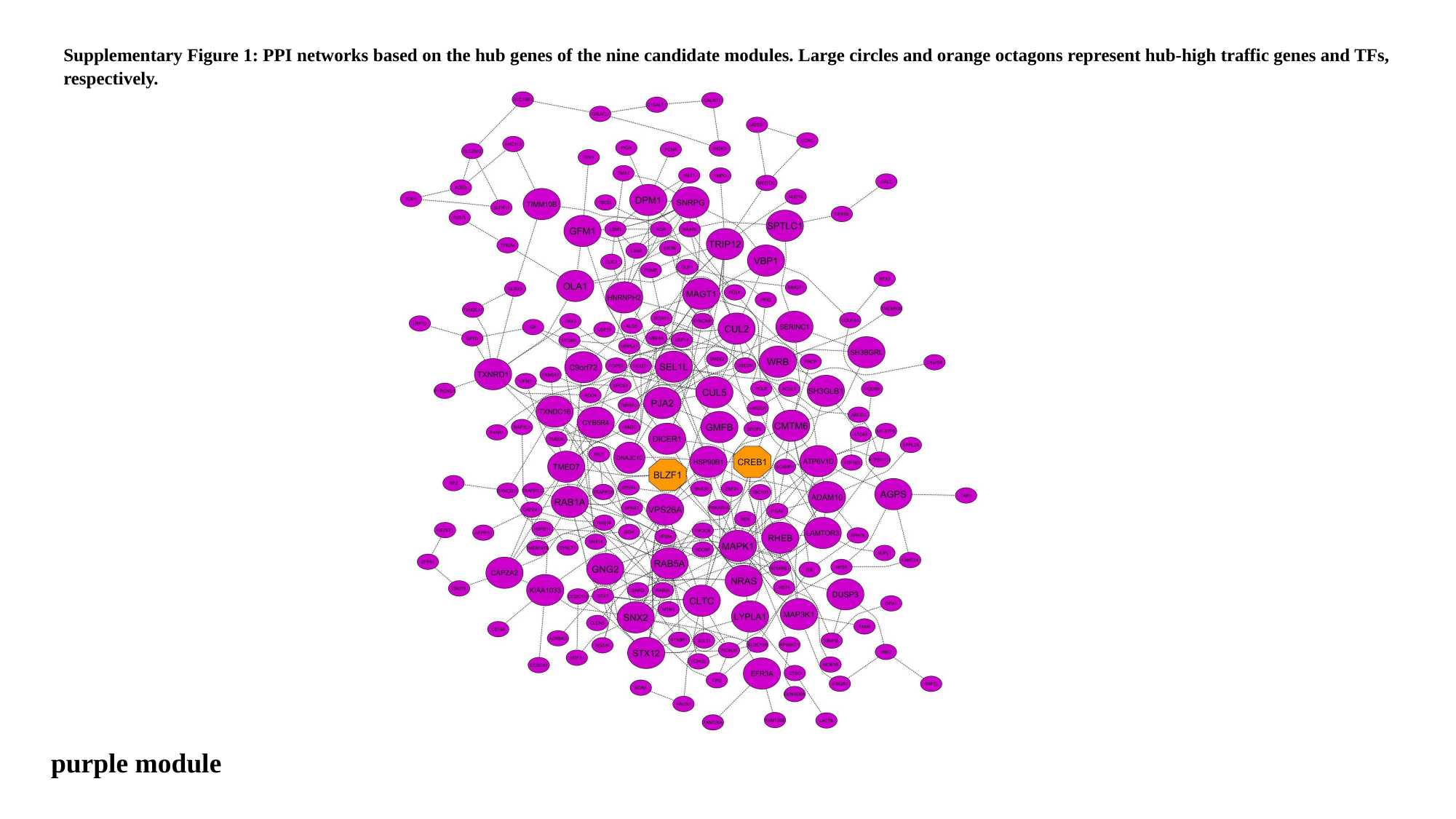

Supplementary Figure 1: PPI networks based on the hub genes of the nine candidate modules. Large circles and orange octagons represent hub-high traffic genes and TFs, respectively.
purple module

## Slide 9
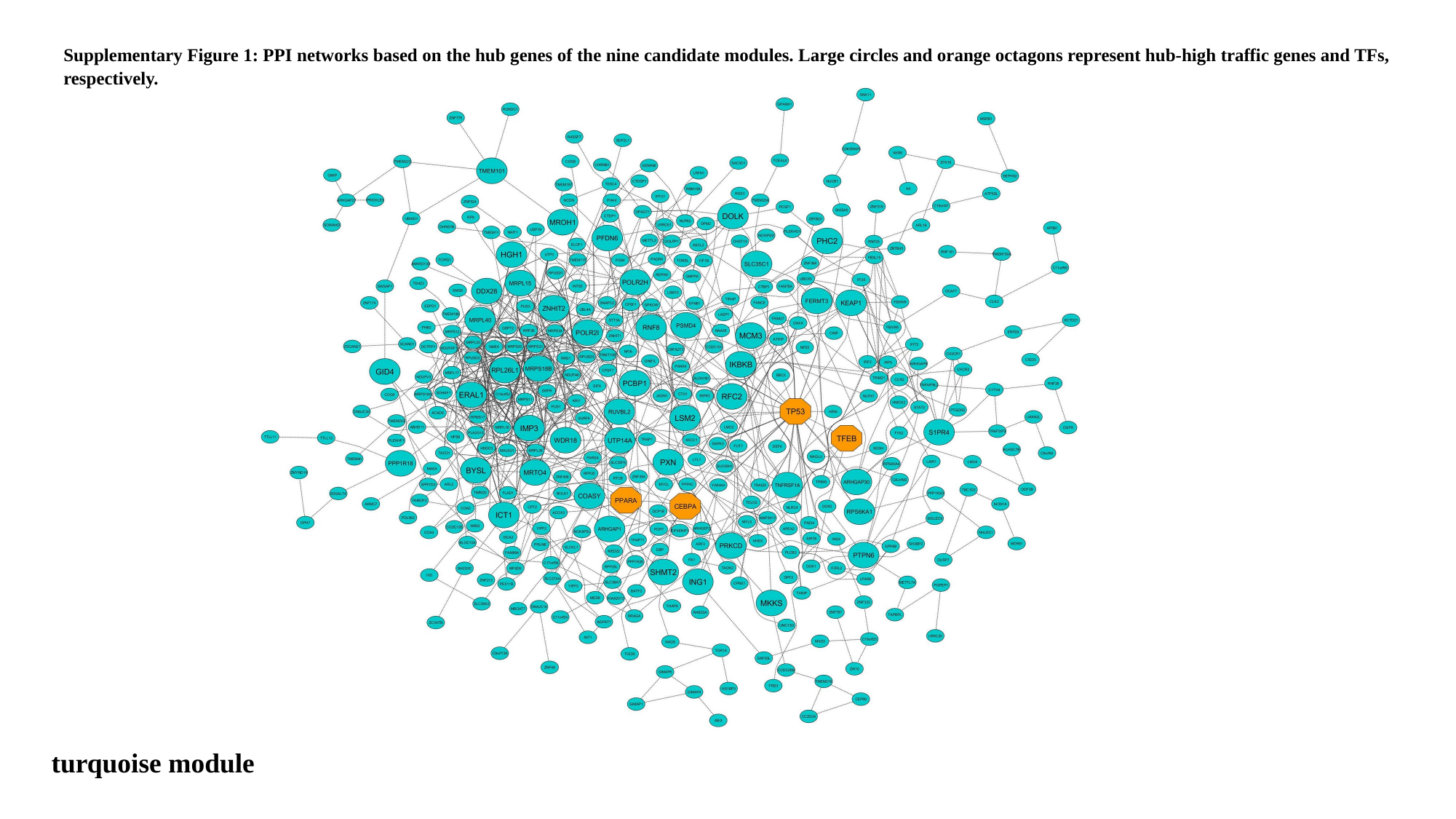

Supplementary Figure 1: PPI networks based on the hub genes of the nine candidate modules. Large circles and orange octagons represent hub-high traffic genes and TFs, respectively.
turquoise module
